# Resurrecting the Activity of the RNA Subunit of Human RNase P

**DOI:** 10.1002/cbic.202500803

**Published:** 2026-03-26

**Authors:** Dan Li, Leif A. Kirsebom, Roland K. Hartmann

**Affiliations:** ^1^ Institut für Pharmazeutische Chemie Philipps‐Universität Marburg Marburg Germany; ^2^ College of Pharmaceutical Sciences Zhejiang University Hangzhou Zhejiang P. R. China; ^3^ Department of Cell and Molecular Biology Biomedical Center Uppsala University Uppsala Sweden

**Keywords:** H1 RNA, human RNase P, ribozyme, ribozyme evolution, tRNA processing

## Abstract

The RNA subunit of human nuclear RNase P, H1 RNA, has low catalytic activity in the absence of its protein cofactors. To improve H1 RNA activity, we introduced minor structural changes toward the bacterial consensus into its catalytic (C) domain. Incorporating a bacterial‐like P4‐J19/4 element increased its ribozyme activity ∼two fold. Changes toward bacterial‐like P2‐P3‐J3/4 or P5‐P15 elements individually impaired its activity, but increased activity ∼five fold when combined with a bacterial P4‐J19/4 element, suggesting that several core elements act cooperatively to enhance the catalytic performance of H1 RNA. Neither fusion of the H1 RNA C‐domain to the bacterial S‐domain nor substrate tethering to H1 RNA resulted in detectable cleavage activity, indicating that low H1 RNA activity is not merely a consequence of defective S‐domain function. Pb^2+^‐probing and UV melting profiles indicate an inherently unstable global structure of H1 RNA that is sensitive to structural alterations. The parental H1 RNA and its two to five fold more active variants could be activated by the *Escherichia coli* RNase P (RnpA) protein at low (10 mM) Mg^2+^ concentration, indicating that bacterial RnpA and eukaryotic Pop5 protein subunits recognize C‐domain core elements common to all RNase P RNAs.

## Graphical abstract

The catalytic RNA (H1 RNA) of the human nuclear tRNA processing enzyme RNase P has >10^5^‐fold lower activity in the absence of its 11 protein cofactors than bacterial RNase P RNAs. By introducing mutations toward the bacterial consensus into its catalytic domain, a two and fivefold activity enhancement could be achieved by changes in the P4‐19/4 region alone and combined with changes in the P2‐3_J3/4 and J4/7_J7/2 regions, respectively.

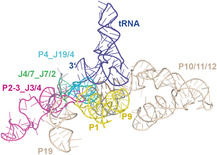

## 1 Introduction

The ubiquitous endoribonuclease P (RNase P) is essential for the 5′‐maturation of precursor tRNA transcripts in all types of cells and organelles. A unique feature of RNase P is its compositional diversity, which includes the ribonucleoprotein complexes consisting of a catalytic RNA subunit and a varying number of proteins (1 in bacteria, 5 in archaea, and 9–10 different in eukarya; for reviews see [1, 2, 3]) as well as protein‐only forms, PRORPs in eukarya and HARPs in bacteria and archaea [4, 5].

Two main architectural types of bacterial RNase P RNA (RPR) have been identified, type A (for ancestral) found in the majority of bacteria, and type B (for *B* *acillus*‐like) being an evolutionary diversification confined to some Bacillota (formerly named Firmicutes). A subgroup of archaeal RPRs show pronounced resemblance to bacterial type A enzymes and were thus classified as type A; others are more simplified versions that are named type M (*M* *ethanococcus*‐like [6]). Archaeal RPRs form a complex with five protein subunits, which have homologs among the 9–10 protein cofactors of eukaryal nuclear RNase P enzymes [7, 8] (for reviews see [1, 2, 3]). The RNA subunits of eukaryal nuclear enzymes are structurally reduced versions of their bacterial counterparts, similar to the archaeal type M RPRs. This change correlates with the increased contribution of proteins (>50%) to enzyme mass in eukarya relative to bacterial enzymes (10%) [9].

Bacterial RNA‐based RNase P enzymes are composed of the catalytic RPR subunit (∼340–400 nt in length, corresponding to 110–130 kDa) and a small single protein cofactor of ≈13 kDa. The RPRs consist of two independently folding domains, the catalytic (C) and the specificity (S) domain [10], the latter interacting with the D/T‐loop “elbow” of tRNA molecules. Since the C‐domain provides all structural elements required for catalysis, bacterial and archaeal RPRs lacking the S‐domain are able to perform catalysis, albeit with catalytic efficiences (*k*_cat_/*K*_M_) reduced by up to four orders of magnitude in the case of bacterial RPRs [11, 12, 13, 14, 15].

RPR subunits from human (H1 RNA) and the lower eukaryote *Giardia lamblia* were demonstrated to have low catalytic activity when analyzed at pH 6.0, conditions at which the chemistry of cleavage is rate‐limiting (see [16, 17, 18, 19, 20]) and metal ion‐induced RNA fragmentation minimized during extended incubation times [21]. The highest activities measured for these eukaryotic RPRs are more than five orders of magnitude lower than those obtained with bacterial RPRs (e.g., measured single‐turnover rate constants at pH 6.0 were *k*_obs_ = 8.4 min^−1^ for *Escherichia coli* RPR versus *k*_obs_ = 3 × 10^−5^ min^−1^ for H1 RNA [21]). Phylogenetic comparative analysis of eukaryal RPRs has identified five conserved regions CRI‐V found also in bacterial and archaeal RPRs [22]. In contrast to bacterial RPRs, most eukaryal homologs have P3 elements that include a large internal loop [22, 23]; furthermore, eukaryal RPRs lack several structural elements present in bacterial type A RPRs, such as P5, P6, and P15‐18 (discussed below), known to be critical for the architecture of RPRs, substrate binding and catalysis [22, 23, 24]. Crosslinking experiments indicated that eukaryal RPRs nevertheless fold into functional structures that specifically bind tRNA even in the absence of protein subunits [25]. A tertiary structure model of eukaryal RPRs was built on the basis of crosslinking results and crystal structures of bacterial RPRs [25]. The model suggested that the eukaryal RPRs contain a core structure similar to that of bacterial RPRs, but lack some structural elements that contribute to catalysis and global stability of the tertiary structure. The cryo‐EM structure of the human RNase P holoenzyme [9] then revealed that H1 RNA acts as a scaffold to which all 11 protein components (including two copies of Rpp30) bind and wrap around the RNA to stabilize its extended single‐helical‐layer conformation. The structure confirmed that accessory RNA elements, essential for substrate binding and catalysis in bacterial RNase P, have been functionally replaced by protein elements in eukaryotic nuclear enzymes.

We previously reported succesfull improvement of the RNA‐alone activity of an archaeal RPR by simultaneously introducing a few strategic changes towards the bacterial type A consensus into the C‐domain (e.g., 435‐fold from *k*_obs_ = 1.7 × 10^−4^ min^−1^ to 740 × 10^−4^ min^−1^ in single‐turnover cleavage kinetics; ref. [26], Table 3 therein). However, the structural changes could only be coaxed into high activity when the aforementioned engineered archaeal C‐domain was tethered to a bacterial S‐domain to replace the native archaeal S‐domain (*k*_obs_ acceleration from 6.7 × 10^−3^ min^−1^ to ≈1 min^−1^; ref. [26], Table 2 therein). Pulukkunat and Gopalan [27] covalently tethered a substrate to an archaeal type M RPR or its isolated C‐domain, both inactive in the *trans*‐processing reaction. These *cis*‐conjugates underwent self‐cleavage in the absence of protein cofactors (*k*_obs_ = ∼0.4 min^−1^ at pH 7, 55°C, 2.5 M NH_4_^+^ and 0.5 M Mg^2+^ for the pre‐tRNA:C‐domain‐only conjugate), demonstrating that even the C‐domain of a structurally minimized type M RPR retains RNA‐alone catalytic potential. Both studies [26, 27] as well as an earlier study by Brown and coworkers [28] provided evidence that the S‐domain of archaeal RPRs is a major determinant for their low RNA‐alone activity, owing to defective substrate positioning by these S‐domains in the absence of protein cofactors. Subsequently, we demonstrated that an engineered archaeal RPR C‐domain can even functionally replace its *E. coli* counterpart in vivo. This adaptation became possible by introducing three minor alterations into the archaeal C‐domain combined with restoration of two interdomain contacts (L9‐P1, L18‐P8) in the chimeric RNA consisting of the archaeal C‐domain and *E. coli* S‐domain [29]. In this context, deletion of the S‐domain in an archaeal RPR lowered RNA‐alone activity ∼17‐fold while activity of the *E. coli* C‐domain without its S‐domain was almost 500‐fold lower than that of the full‐size RPR [15]). This indicates that the S‐domain of archaeal RPRs supports the function of the C‐domain to a much lesser extent than in bacterial RPRs.

Here we explored if the RNA‐alone activity of human RPR (H1 RNA) could be improved by introducing mutations toward the bacterial consensus structure into its C‐domain, by fusion of the catalytic domain to the S‐domain of *E. coli* RPR, or by tethering a substrate RNA covalently to H1 RNA. Only the first‐mentioned approach resulted in activity increases, for which the introduction of a bacterial P4‐J19/4 element played an instrumental role in both, RNA‐alone reactions in high salt buffer (800 mM NH_4_^+^, 160 mM Mg^2+^) or when supplemented with the *E. coli* RnpA protein in low salt buffer (150 mM NH_4_^+^, 10 mM Mg^2+^) where the RNA alone is inactive. The mutational and kinetic analyses as well as Pb^2+^‐probing and UV melting profiles indicate that the structure of H1 RNA in the absence of its native protein cofactors is inherently unstable though globally optimized, making the RNA's activity highly sensitive to structural alterations.

## 2 Results

### 2.1 Design of H1 RNA Variants

**FIGURE 1:**
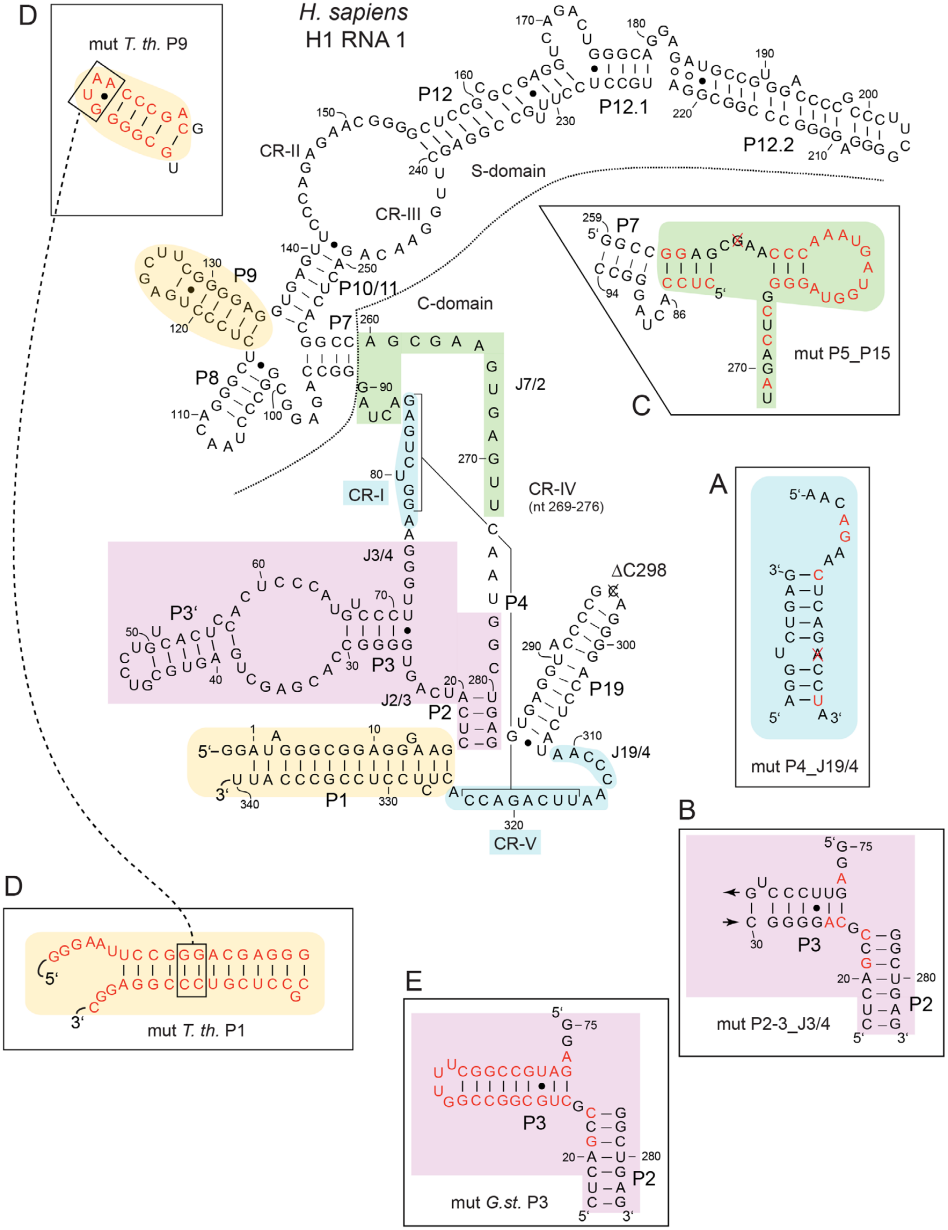
Secondary structures of H1 RNA variants in the presentation introduced by James et al. (1988) [30]. *P* = paired sequence elements, numbered from the RNA's 5′‐ to 3′‐end; J = joining sequences connecting two helical elements, e.g., J7/2 indicating the single‐stranded sequence segment connecting helices P7 and P2; CR‐I to CR‐V: conserved regions in RPRs. The nucleotide sequence is based on the original H1 RNA sequence (NCBI Nucleotide Sequence: NR_002312.1; or GenBank: S68670.1) except for the absence of a C residue at the top of P19 (crossed out C, marked as ΔC298) and a U to C exchange at position 323. Historically, this variant was termed H1 RNA Δ298C325 [21]; we have retained this nomenclature (see Table 1) although Δ298C325 is Δ297C323 according to our numbering system. The secondary structure has been adopted from ref. [9]. A difference to the H1 RNA Δ298C325 structure presented in ref. [21] is the presence of a bulged A residue at position 3 in helix P1 as encoded in the genome sequence. Our parental “H1 RNA Δ298C325” is termed H1 RNA 1 for simplification. H1 RNA variants also had two extra G residues at the 5′‐end for reasons of T7 transcription efficiency and a GAAUU extension at the 3′‐end (not shown) derived from the EcoRI site used for plasmid linearization. Regions subjected to mutation in the presented study are highlighted by different colors. Sequences and predicted structures of the mutated elements (indicated by the prefix “mut”) are given in the boxes around the secondary structure, with altered nucleotides colored in red and deleted nucleotides crossed out, and nucleotide numbering according to that of the parental H1 RNA 1.

The H1 RNA variant Δ298C325 was previously shown [21] to have higher catalytic activity than variants Δ298U325, C298U325, and C298C325; the latter two were reported in the literature as natural variants of H1 RNA. Variant Δ298C325, considered to be instrumental in the initial detection of H1 RNA‐alone cleavage activity [21], was also selected as the parental RNA in the present study for reasons of comparability with the aforementioned study (variant Δ298C325 is named H1 RNA 1 in the following; for details, see Figure 1). The reason for its somewhat higher activity compared with the other aforementioned sequence variants is not clear, but is likely related to RNA folding.

In our reverse‐engineering approach, several changes toward the bacterial consensus (Figure S1) were introduced into H1 RNA 1. The constructed H1 variants and their structural alterations are illustrated in Figure 1 and also in the context of the secondary structure model (Figure S2) more oriented towards the RPR tertiary structure [31]; the altered structural elements are further illustrated within the 3D structure of H1 RNA (Figure S3) extracted from the human cryo‐EM holoenzyme structure in complex with tRNA [9]. The reasons for choosing these changes are briefly summarized below and more details are provided in the Supporting Information.

Mutations **A** (mut P4_J19/4; resulting in H1 RNA 5) introduced a bacterial‐like helix P4 and junction J19/4. Both elements in the core of the C‐domain are crucial for stabilizing the functional conformation of the RNA [32, 33], coordination of divalent metal ions [34, 35, 36], as well as substrate binding and catalysis [37, 38, 39, 40, 41, 42].

Mutations **B** (mut P2‐3_J3/4. H1 RNA 2) were designed to restore a bacterial‐like P2‐P3 subdomain. The P2/P3 region, located at the bacterial RnpA protein binding surface, is part of the catalytic core [24, 26, 43, 44]. Extending P2 from 6 to 7 bp and reducing J2/3 to a single G residue increased RNA‐alone activity of an archaeal RNA 135‐fold [26].

Mutations **C** (mut P5/P15, H1 RNA 4) introduced P5 and P15 and flanking sequences that are found in type B bacterial RPRs. The two modules positioned in the core structure are generally important for the overall conformation of bacterial RPRs, binding of substrates via the 3′‐CCA end, Mg^2+^ binding and RnpA interaction [24, 45, 46, 47, 48] (for a recent review see [3]). The absence of P5 and P15 was also considered to contribute to the structural instability of the H1 RNA core structure.

We further constructed variants H1 RNA 3 (mutations **A** + **B**) and H1 RNA 6 (mutations **A** + **B** + **C**) to monitor the effect of combined structural changes.

Introduction of the P1 and P9 modules of *Thermus thermophilus* RPR into H1 RNA (mutations **D,** mut *T. th.* P1/P9, H1 RNA 7) aimed at implementing this interdomain tertiary contact to support interdomain orientation and hence RPR function. Establishing this contact in a chimera consisting of an archaeal C‐domain and *E. coli* S‐domain was a final alteration required to restore survival of a conditionally lethal *E. coli* RNase P depletion strain [29].

With mutations **E** (mut *G. st.* P3, variant H1 RNA 8)**,** we also removed the internal loop and distal part of the eukaryal P3 subdomain to install an entirely bacterial‐type P2‐3_J3/4 region.

### 2.2 Functional Analysis of H1 RNA Variants in RNA‐Alone Reactions

**FIGURE 2:**
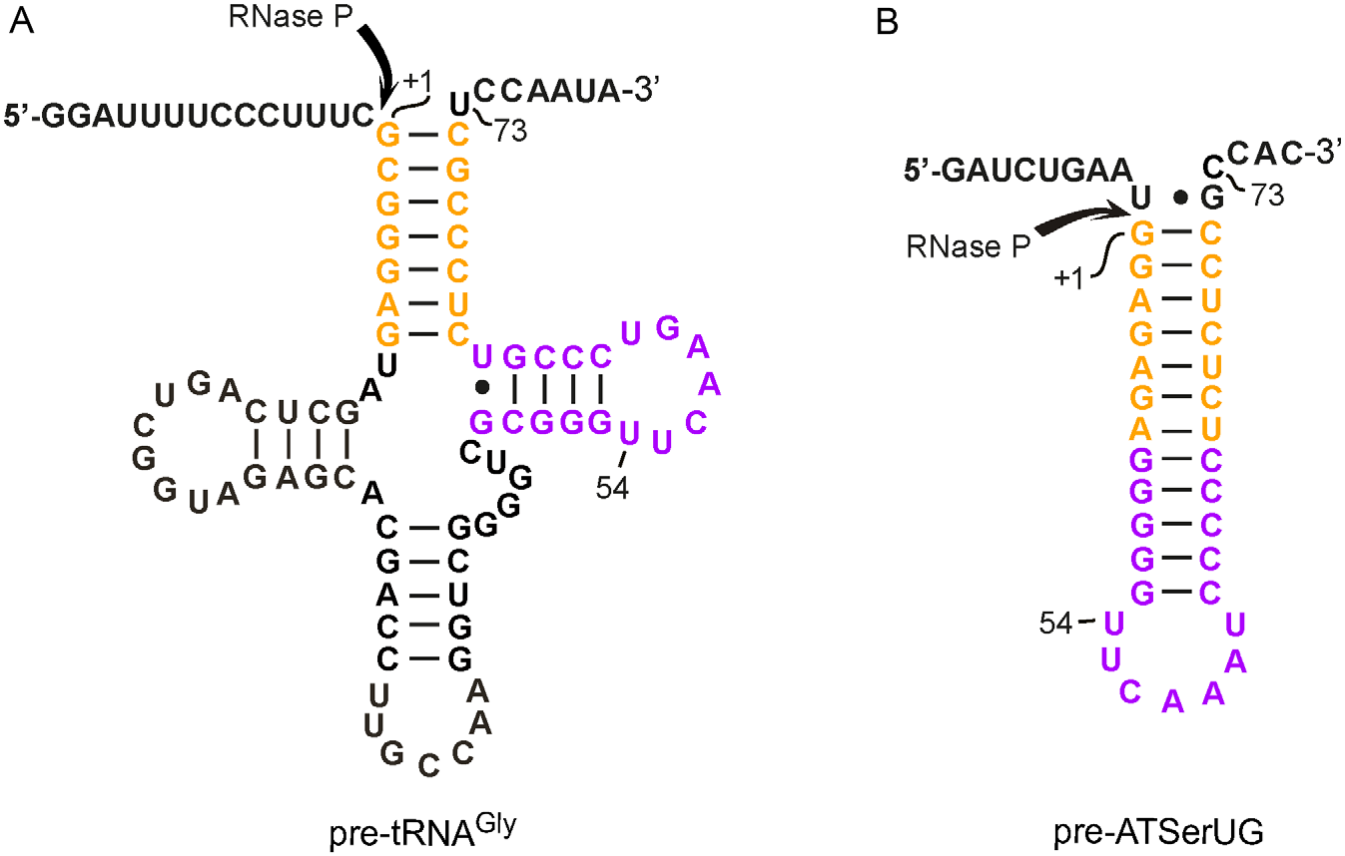
Secondary structures of substrates ptRNA^Gly^ and pATSerUG. The canonical RNase P cleavage sites are marked with arrows. The acceptor‐stem and T‐arm modules of the two structures are colored correspondingly.

**FIGURE 3:**
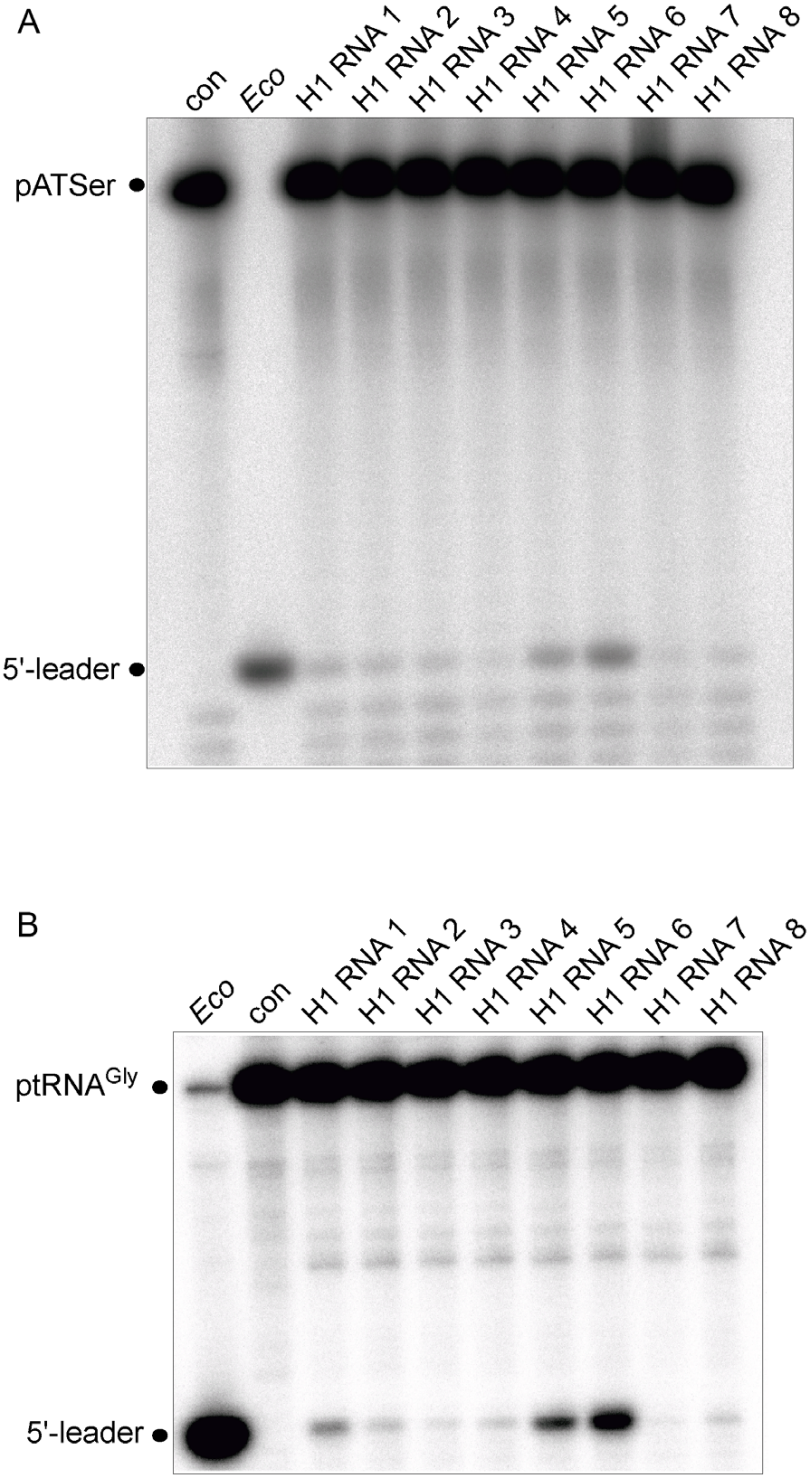
Cleavage of (A) pATSerUG and (B) ptRNA^Gly^ by H1 RNA variants specified in Table 1 in the absence of any protein cofactors. Lanes con: no RPR present; lanes *Eco*: assay with *E. coli* RPR. 5′‐[^32^P]‐labeled substrates (approximately 5 nM, 40 000–50 000 Cherenkov cpm per reaction and gel lane) were incubated with H1 RNA variants (6 µM) or *E. coli* RPR (370 nM) in buffer A (50 mM MES pH 6.0 [37°C], 800 mM NH_4_OAc, 160 mM Mg[OAc]_2_) for 22 h at 37°C (*E. coli* RPR for 30 min at 37°C); control samples without RPR (lanes con) were also incubated for 22 h at 37°C. Reactions were analyzed by 22% (w/v) denaturing PAGE (7 M urea). For details, see Experimental Section.

The H1 RNA variants (see above) were tested for RNA‐alone activity with two substrates, precursor tRNA^Gly^ (ptRNA^Gly^) and pATSerUG (a well characterized hairpin model substrate; Figure 2; also used in [21]). H1 RNA 1 showed low but significant RNA‐alone activity (Figure 3 and Table 1) compared to the reaction without enzyme (lane “con”), in agreement with Kikovska et al. [21]. The activity increased ∼five fold for H1 RNA 6 (mutations **A** + **B** + **C**) and ∼two fold for H1 RNA 5 (mutations **A**) with both substrates. All other variants displayed catalytic activities somewhat lower than H1 RNA 1 (Table 1): H1 RNA 2 (mutations **B**), H1 RNA 3 (mutations **A** + **B**), H1 RNA 4 (mutations **C**), H1 RNA 7 (mutations **D**) and H1 RNA 8 (mutations **E**). This suggests a cooperative effect caused by the combined changes **A, B,** and **C**. Changes **B** or **C** alone and also combination of changes **A** + **B** (H1 RNA 3) failed to improve activity (Table 1).

**TABLE 1:** Processing rates of H1 and HE RNA variants in RPR‐alone reactions using ptRNA^Gly^ or pATSerUG as substrate.

	*k*_obs_, min^−1^	
	pATSerUG	ptRNA^Gly^
H1 RNA 1 (Δ298C325)	1.1 (±0.2) × 10^−6^	1.4 (±0.3) × 10^−5^
H1 RNA 2: mut P2‐3_J3/4 (*mutations* **B**)	6.0 (±0.7) × 10^−7^	3.4 (±0.1) × 10^−6^
H1 RNA 3: mut P2‐4_J4/19 (*mutations* **A + B**)	5.3 (±0.2) × 10^−7^	1.7 (±0.1) × 10^−6^
H1 RNA 4: mut P5_P15 (*mutations* **C**)	2.2 (±0.5) × 10^−7^	2.0 (±0.2) × 10^−6^
H1 RNA 5: mut P4_J19/4 (*mutations* **A**)	2.4 (±0.1) × 10^−6^	2.8 (±0.1) × 10^−5^
H1 RNA 6: mut P2‐5_P15_J19/4 (*mutations* **A + B + C**)	5.8 (±0.1) × 10^−6^	7.0 (±0.1) × 10^−5^
H1 RNA 7: mut P1_P9 (*T. th* P1, P9, *mutations* **D**)	5.4 (±0.9) × 10^−7^	1.6 (±0.1) × 10^−6^
H1 RNA 8: mut P3 (*G. st* P3, *mutations* **E**)	5.1 (±0.8) × 10^−7^	2.3 (±0.2) × 10^−6^
HE RNA 1 (*E. coli* S‐domain)	n.d.	n.d.
HE RNA 3 (*E. coli* S‐domain, *mutations* **A + B**)	n.d.	n.d.
HE RNA 5 (*E. coli* S‐domain, *mutations* **A**)	n.d.	n.d.
HE RNA 6 (*E. coli* S‐domain, *mutations* **A + B + C**)	n.d.	n.d.

H1 RNAs 2–8 are derivatives of H1 RNA 1 (Δ298C325); the introduced mutations are illustrated in Figure 1; chimeric HE RNAs variants deviate from the corresponding H1 RNA variant by carrying the S‐domain of *E. coli* RPR (see Figure 5); n.d.: not detectable. For further details, see Experimental Section; *k*_obs_ values are mean values (± standard deviation) based on three individual experiments.

### 2.3 Mg^2+^ Dependence of Cleavage by H1 RNAs 5 and 6

**FIGURE 4:**
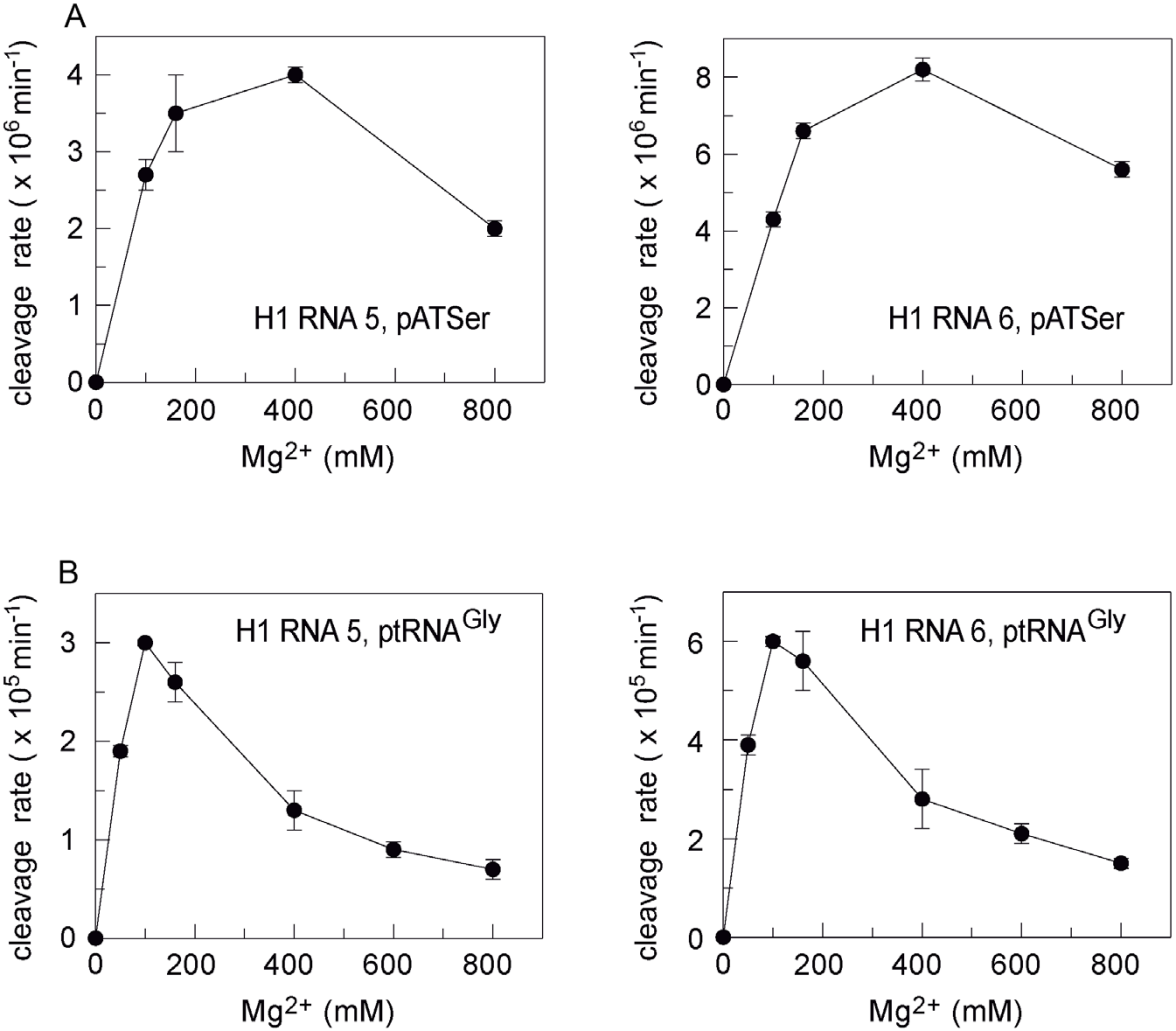
Rates for cleavage of (A) pATSerUG and (B) ptRNA^Gly^ by H1 RNAs 5 and 6 at varying Mg^2+^ concentrations. Assays were conducted for 22 h at 37°C at 6 µM H1 RNA, approximately 5 nM 5′‐[^32^P]‐labeled substrate, 50 mM MES pH 6.0 [37°C], 800 mM NH_4_OAc, and indicated Mg[OAc]_2_ concentrations. For further details, see legend to Figure 3. Data are based on three independent experiments; errors: standard errors of the mean.

For our most active variants H1 RNA 5 and 6, we analyzed the Mg^2+^ dependence of RNA‐alone cleavage of pATSerUG and ptRNA^Gly^. For H1 RNA 1, a concentration of 400 mM Mg^2+^ was previously found to be optimal for cleavage of pATSerUG, and higher concentrations decreased activity [21]. A similar Mg^2+^ dependence was observed for pATSerUG cleavage by H1 RNAs 5 and 6 (Figure 4A), suggesting that the modest activity improvements of these variants compared with the parental H1 RNA 1 had no substantial effect on the overall Mg^2+^ dependency.

The Mg^2+^ profiles with ptRNA^Gly^ differed significantly from those observed with pATSerUG, indicating differences in the binding modes of the two substrates. Mg^2+^ optima for ptRNA^Gly^ cleavage by H1 RNAs 5 and 6 were at 100 mM, and activity decreased ∼four fold at 800 mM Mg^2+^ (Figure 4B). The decrease in activity above 100 mM Mg^2+^ might be explained by assuming that all activating Mg^2+^ binding sites are occupied at 100 mM, and inhibitory Mg^2+^ binding sites are increasingly occupied at [Mg^2+^] > 100 mM, which counteract the effect of the activating Mg^2+^ ions bound at other sites. Correspondingly, the occupancy of activating binding sites might be shifted to higher [Mg^2+^] for cleavage of pATSerUG; as a consequence, the effect of inhibitory Mg^2+^ ions manifests only at higher Mg^2+^ concentrations (800 mM). An alternative explanation for the declining activity at Mg^2+^ concentrations above the optima (Figure 4) could be an unfavorable structural tightening and lower conformational dynamics of substrates at higher Mg^2+^ concentrations. The sharper optimum at lower Mg^2+^ concentration (100 mM) for ptRNA^Gly^ versus pATSerUG would be consistent with overall stronger Mg^2+^ binding sites at the H1 RNA‐ptRNA^Gly^ interface relative to those at the H1 RNA‐pATSerUG interface.

The rate constants for cleavage of pATSerUG by H1 RNAs 5 and 6 were roughly 10‐fold lower than the corresponding ones for ptRNA^Gly^ relative to pATSerUG (Figure 4). Apparently, productive E–S complex formation is impaired for the truncated hairpin substrate (pATSerUG). The latter substrate lacks the D‐loop and cannot form the complete stacking interaction between two nucleotides in the H1 RNA CR‐II/CR‐III region and tRNA residues 19 and 56 [9]. In addition, pATSerUG has a rigid continuous 12‐bp Watson–Crick stem while the ptRNA can form the natural slight kink between acceptor‐ and T‐stem. This feature was found to be crucial for substrate recognition by the nuclear human and *Xenopus laevis* RNase P enzymes [49, 50] and may have also impacted the RNA‐alone reactions studied here.

**FIGURE 5:**
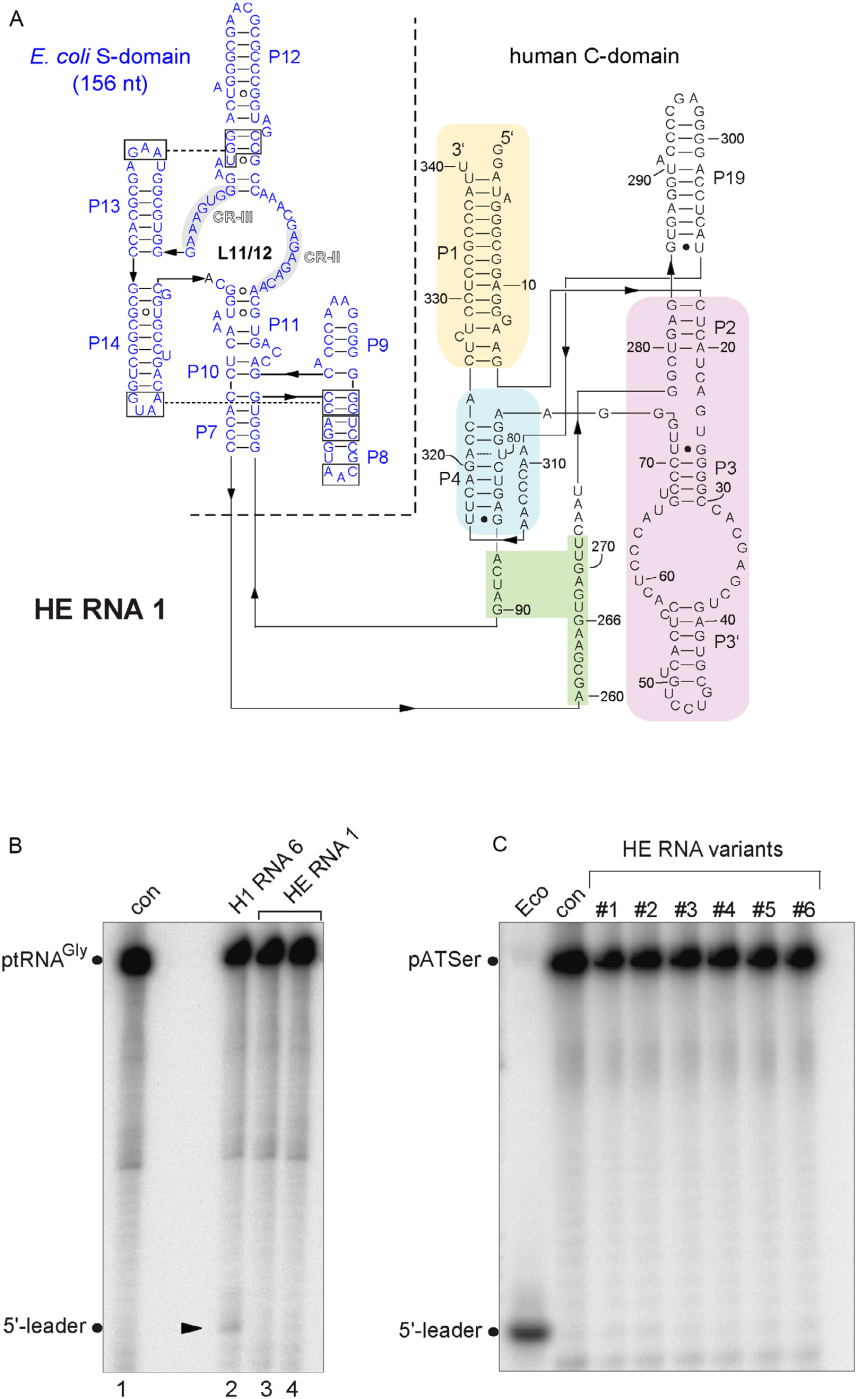
Secondary structure and activity of HE chimeras. (A) The S‐domain from *E. coli* (blue letters) was fused to the C‐domain (black letters) of H1 RNA 1 (see Figure 1). (B) Cleavage assays with HE RNA 1 (lanes 3 and 4, replicate samples) next to H1 RNA 6 (lane 2), using ptRNA^Gly^ as substrate; lane con: without enzymatic RNA. (C) Cleavage assays with HE variants using pATSerUG as substrate; lane *Eco*: assay with *E. coli* RPR; lane con: incubation without RPR. Assays were performed as described in Figure 3 for H1 RNA variants and *E. coli* RPR. HE RNAs 3, 5, and 6 carry the same changes in the C‐domain as H1 RNAs 3, 5, and 6 (see Figure 1).

**FIGURE 6:**
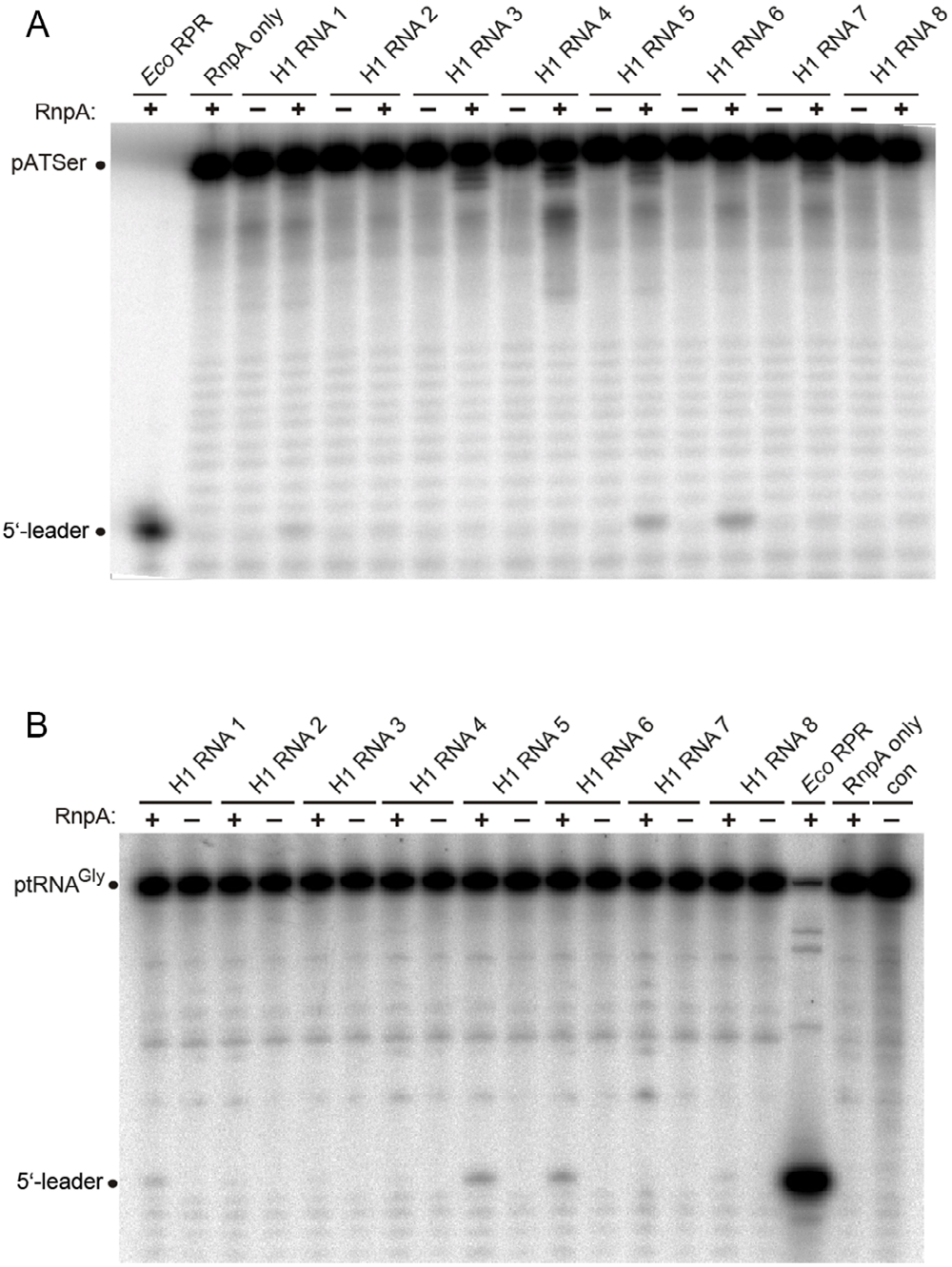
Cleavage of (A) pATSerUG and (B) ptRNA^Gly^ by H1 RNA variants in the presence of the *E. coli* RnpA protein. Reaction mixtures were incubated for 20 min (*Eco* RPR) or 4 h (H1 RNA variants) at 37°C and analyzed by 22% (w/v) denaturing (7 M urea) PAGE. Lanes “RnpA only”: no RPR added; lane “con”: no protein and RPR added. The latter control samples were also incubated for 4 h at 37°C. For details of cleavage assays, see Experimental Section.

In single‐turnover processing reactions of different substrates (including full‐length pre‐tRNA and pATSer derivatives) catalyzed by *E. coli* RPR under the same conditions as in the present study, Mg^2+^ optima of 300–400 mM or larger were observed as well (ref. [51], Figures 5 and 6 therein). Hence, the [Mg^2+^] optimum for cleavage of pATSerUG appears not specific to H1 RNA and its variants, and the sharp optimum at 100 mM Mg^2+^ for ptRNA^Gly^ cleavage by H1 RNAs 5 and 6 might be an idiosyncrasy of this particular ptRNA substrate. Based on these findings, conclusive evidence is lacking that the higher [Mg^2+^] optimum for pATSerUG versus ptRNA^Gly^ cleavage (Figure 4) might reflect a compensatory effect owing to the lower H1 RNA affinity for the pATSer substrate.

### 2.4 Human–Bacterial RPR Chimeras

We constructed a chimera consisting of the *E. coli* S‐domain and the C‐domain of H1 RNA 1 (chimera HE RNA 1, Figure 5A) to improve substrate affinity and positioning, a strategy that was successful in efforts to activate archaeal RPR C‐domains [26, 27, 29]. In addition, mutations **A**, **A + B**, and **A + B + C** were introduced into HE RNA, generating variants HE RNA 5, 3, and 6, respectively. However, all chimeric HE RNA variants failed to process pATSerUG or ptRNA^Gly^ above background (Figure 5B,C).

### 2.5 Tethered H1 RNA‐Substrate Constructs

Substrate tethering to RPR was developed to strengthen substrate binding and thereby cleavage efficiency, and to investigate the catalytic mechanism in *cis*‐cleavage reactions [27, 35, 52, 53]. The archaeal type M *Methanocaldococcus jannaschii* RPR was reported to be catalytically inactive in the normal *trans*‐cleavage reaction in the absence of its protein cofactors. However, a ptRNA covalently tethered to this RPR was efficiently cleaved in *cis* [27]. We followed this strategy and designed corresponding H1 RNA‐substrate conjugates, H1‐pATSerUG‐5 and H1‐pATSerUG‐3 (Figure S4A,B). In these conjugates, the single‐stranded nucleotides between P7 and P2 were opened, and the pATSerUG substrate was linked either to the 3′‐ or 5′‐end at this breakage point in H1 RNA. The attachment site is at the position where the L15 loop is located in bacterial RPR, and thus being part of the substrate binding interface (see above). Comparable constructs were previously shown to be active in *cis*‐cleavage reactions [27, 52]. An additional single‐stranded segment was inserted (nt in the gray‐shaded area 3′ of the pATSer hairpin in panel A or 5′ in panel B; Figure S4) at the attachment site to provide sufficient flexibility for substrate docking to the active site. The natural H1 RNA 5′‐ and 3′‐ends were connected via a loop in these circularly permuted constructs (Figure S4). Considering the possibility that the overall structure might be too disordered in constructs H1‐pATSerUG‐5 and ‐3, we designed a third conjugate, H1‐P15‐pATSerUG‐5. Here, a 4‐bp long P15 helix was introduced to rigidify the attachment site of pATSerUG in H1 RNA (Figure S4C). None of the enzyme‐substrate conjugates underwent detectable *cis*‐cleavage above background under the tested high salt conditions (800 mM NH_4_^+^, 160 mM Mg^2+^, Figure S5). It may be worth testing additional variants in future experiments, including ones containing full‐length tRNAs and alternative substrate attachment sites and linkers, possibly combined with in vitro selection approaches [54] to enrich potentially self‐cleaving variants.

### 2.6 Activity of H1 RNA Variants Under Low Salt Conditions in the Presence of the *E. coli* RnpA Protein

All H1 variants were also analyzed for catalytic activity in the presence of the *E. coli* RnpA protein under low salt conditions (150 mM NH_4_^+^, 10 mM Mg^2+^) where RNA‐alone activity is not detectable. RnpA‐dependent processing was observed for H1 RNA 1, 5, and 6 (Figure 6), the variants that also showed the highest activity in the RNA‐alone reaction at high salt (800 mM NH_4_^+^, 160 mM Mg^2+^). *E. coli* RnpA protein incubated with substrate alone under the same conditions failed to produce any cleavage product, ruling out an RPR contamination in the RnpA protein preparation. Our findings document that H1 RNA 1 can productively interact with the *E. coli* RnpA protein, consistent with a previous report [55].

Chimeric HE RNA variants 1, 3, 5, and 6 (Figure 5) failed to catalyze detectable *trans*‐cleavage of substrates pATSerUG or ptRNA^Gly^ in the presence of the *E. coli* RnpA protein (data not shown), possibly due to misfolding of the C‐domain in the presence of the heterologous S‐domain.

### 2.7 Structural Probing of Selected H1 RNA Variants

**FIGURE 7:**
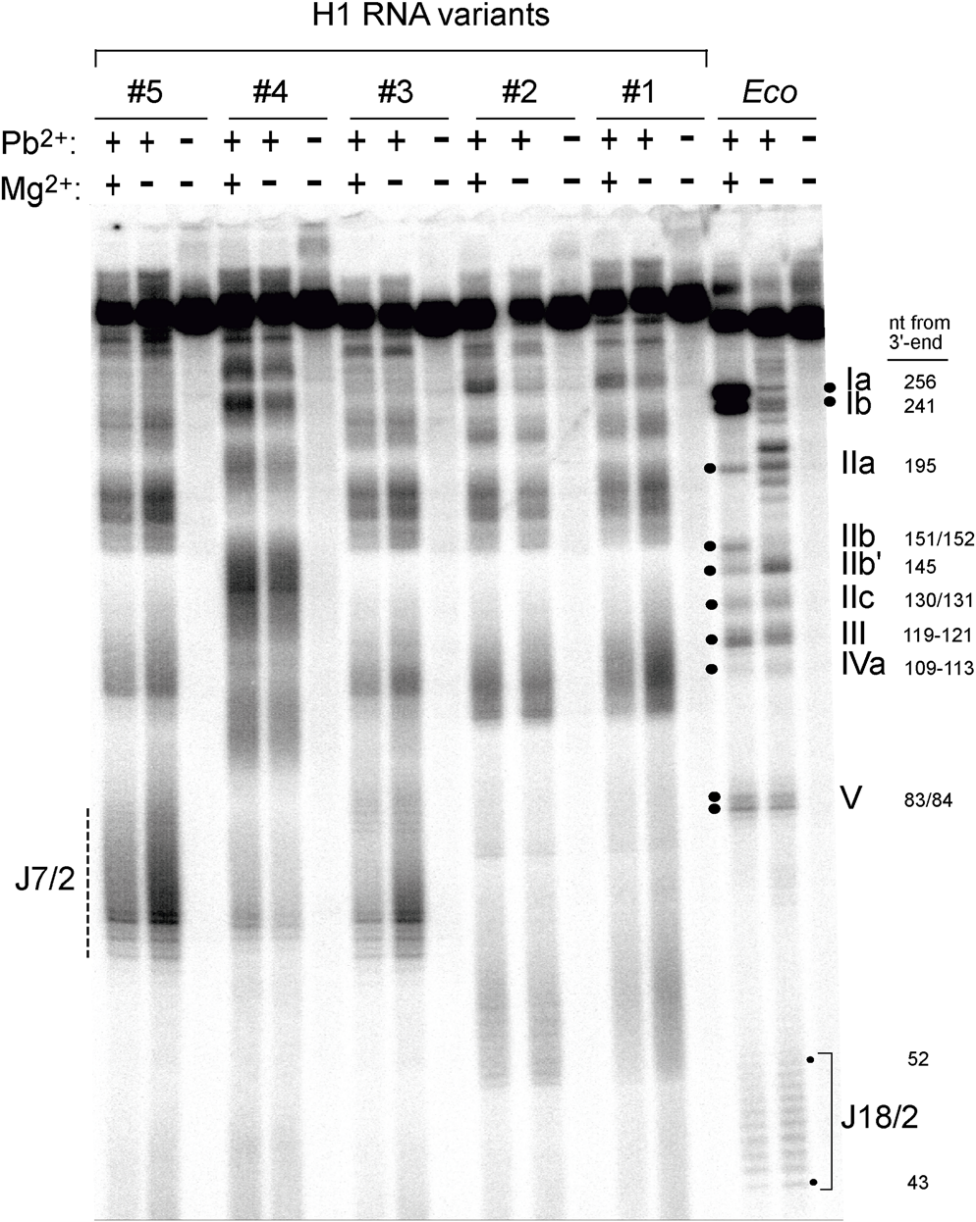
Pb^2+^‐induced hydrolysis of H1 variants next to *E. coli* RPR (*Eco*); RNAs were 3′‐[^32^P]‐endlabeled. P RNAs (250 nM including trace amounts [20 000  Cherenkov cpm] of 3′‐endlabeled RNA) were preincubated in 50 mM Tris‐HCl pH 7.5, 100 mM NH_4_Cl, with or without 2 mM Mg(OAc)_2_, for 2 min at 70°C and then for 10 min at 37°C. Hydrolysis was initiated by adding freshly dissolved Pb(OAc)_2_ solution to a final concentration of 0.5 mM. The reactions were performed at 37°C for 6 min, terminated by mixing with loading buffer (10 M urea, 10 mM EDTA, 2% [w/v] bromophenol blue), and analyzed on 8% (w/v) PAA (7 M urea) sequencing gels. Samples without Mg(OAc)_2_ and Pb(OAc)_2_ were used as controls. The dashed vertical line on the left depicts the region of increased Pb^2+^ accessibility, a hallmark of the variants carrying mutations **A**; based on the known position of prominent Pb^2+^ cleavage sites in *E. coli* RPR, this region was tentatively assigned to J7/2 of H1 RNAs 3 and 5. Prominent Pb^2+^‐hydrolysis sites in *E. coli* (*Eco*) RPR (for references see main text) are marked at the right margin together with the nt length of the 3′‐endlabeled cleavage fragments. For illustration of the location of Pb^2+^‐induced hydrolysis sites in the structure of *E. coli* RPR, see Figure S6. *Eco* RPR was transcribed with T7 RNA polymerase from plasmid pJA2 linearized with FokI, yielding a 377‐nt transcript with native 5′‐ and 3′‐ends [58]. After 3′‐endlabeling with 5′‐[^32^P]‐pCp, the RNA had a length of 378 nt. The lengths of T7‐transcribed H1 RNA variants were: H1 RNA 1, 347 nt; H1 RNA 2, 349 nt; H1 RNA 3, 349 nt; H1 RNA 4, 366 nt; and H1 RNA 5, 347 nt; for further details on H1 RNA variants, see legend to Figure 1.

The solution structures of H1 RNA 1–5 were probed by Pb^2+^‐induced hydrolysis, which is specific for single‐stranded, flexible RNA regions, as well as high affinity metal ion binding sites in RNA molecules [56, 57]. Pb^2+^‐hydrolysis patterns of H1 RNA variants reproducibly had a more diffuse appearance than those for *E. coli* RPR (*Eco*; Figure 7), and we were unsuccessful in obtaining clear RNase T1 cleavage ladders for accurate cleavage site assignments. Nonetheless, the cleavage patterns are informative, as they differed from that of *E. coli* RPR: (i) susceptible regions were more extended, suggesting an increase in the proportion of flexible regions and less defined boundaries between single‐stranded and helical segments; (ii) no prominent Pb^2+^‐cleavage sites attributable to high affinity metal ion binding sites, such as sites Ia and Ib in *E. coli* RPR (Figure 7), were observed for the analyzed H1 variants; (iii) compared with *E. coli* RPR, the amount of Mg^2+^‐assisted structure formation is lower for H1 RNA variants, as inferred from the only minor changes in the Pb^2+^‐hydrolysis patterns upon addition of Mg^2+^; (iv) mutations **A** (present in H1 RNA 3 and 5) and **C** (H1 RNA 4) caused idiosyncratic global changes in the Pb^2+^‐hydrolysis patterns relative to H1 RNA 1, suggesting that the RNA's global structure is sensitive to structural alterations and thus inherently unstable; and (v) finally, *E. coli* RPR (*Eco*, 377 nt) is larger than any of the H1 RNA variants (∼347–366 nt), but migrated faster than all H1 variants in denaturing polyacrylamide gels (Figure 7). A similar observation was made by Gold et al. [59] who inferred an H1 RNA length of 400 nt from migration in 8% denaturing PAGE. The actual length was later identified to be about 340 nt [60], based on co‐electrophoresis of H1 RNA with single‐stranded DNA markers in 5% denaturing PAA gels [61]. These and our findings may either reflect an unfolded extended state of H1 RNA and its variants or the possibility that the expanded P12 element of H1 RNAs may persist during electrophoresis and form a protuberance that retards gel mobility in 8% denaturing PAA gels.

Further differences between individual H1 variants include faster gel migration of full‐length H1 RNAs 2 and 3 relative to variants 1, 4, and 5. The common feature of H1 RNAs 2 and 3 is a bacterial version of P2/P3. The reinforced P2/P3 elements may resist complete denaturation during electrophoresis, resulting in a more compact structure that migrates faster. H1 RNA 3 and 5 displayed similar Pb^2+^‐hydrolysis patterns, which differed from that of H1 RNA 1. They both contain mutations A (mut P4_J19/4; Figure 1). These mutations in the P4 region resulted in increased susceptibility to Pb^2+^‐induced hydrolysis in the region marked with a dashed line (Figure 7, left margin). Taking the prominent and well documented [15, 51, 56, 62, 63, 64] Pb^2+^‐hydrolysis sites of *E. coli* RPR as a reference (see Figure S6) for tentative size assignment, this region should correspond to the extended J7/2 region of H1 RNA (see Figure 1), expected to be accessible to Pb^2+^‐induced hydrolysis. In contrast, H1 RNAs 1 and 2 were susceptible in the region approximately 50–60 nt from the 3′‐end where P19 is predicted to form. This finding would imply that P19 is unfolded in these variants, although further experiments are required to verify this possibility.

### 2.8 UV Melting Profiles of H1 RNA Variants

**FIGURE 8:**
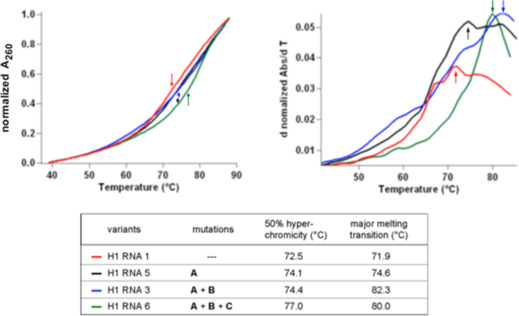
Representative UV melting profiles of H1 RNAs 1, 3, 5, and 6. Graph on the left: melting curves of the four H1 RNA variants recorded at 260 nm in the presence of 250 nM RPR in a buffer containing 50 mM MES pH 6.0, 2 mM EDTA, 100 mM NH_4_OAc, and 4.5 mM Mg(OAc)_2_; hyperchromicity values were normalized to 0.0–1.0 (normalized A_260_); normalized melting curve shapes were not affected by variation of RPR concentration, indicating that intramolecular unfolding was measured. The curves shown represent the mean of at least three measurements, and the colored arrows mark the approx. temperatures at which 50% hyperchromicity was achieved (72.5°C for H1 RNA 1, 74.1°C for H1 RNA 5, 74.4°C for H1 RNA 3°C, and 77°C for H1 RNA 6; summarized in the bottom table). Graph on the right: corresponding first derivatives of the melting curves (d normalized Abs/dT). The major melting transitions (corresponding to *T*_m_ values in the case of simpler systems such as the melting of DNA oligonucleotide duplexes) are indicated by arrows and are summarized in the bottom table. For more experimental details, see “UV melting analyses” in the Supporting Information.

UV melting profiles were analyzed for H1 RNAs 1, 3, 5, and 6 in the presence of 4.5 mM Mg^2+^ and 100 mM NH_4_^+^ (Figure 8) as in our previous study on variants of archaeal RPR from *M. thermoautotrophicus* [26]. For H1 RNA 1, the first derivative curve revealed multiple melting transitions, one major transition appearing at 71.9°C (Figure 8, right panel and tabled below the graphs); the 50% hyperchromicity point of the denaturation curve (between 37°C and 90°C; Figure 8, left panel) was at ∼72.5°C; the latter value increased to about 74.1°C for H1 RNA 5 and 74.4°C for H1 RNA 3, and to ∼77°C for H1 RNA 6. These values are consistent with the gradual overall stabilization of the RNA structure by mutations **A**, **A + B** and finally **A + B + C** (H1 RNA 6). Based on the first derivative of the melting profile (d*A*_260_/d*T*; Figure 8, right panel), H1 RNA 6 was the only variant with a single major unfolding transition at high temperature. H1 RNA 3, and to a lesser extent H1 RNA 5, show increased unfolding in the range of approximately 50°C–60°C. A common feature of H1 RNA 1 and 5 is the broad overlap of multiple folding transitions between ∼65°C and 85°C. The rugged first derivative curves of H1 RNAs 1, 3 and 5 may report the coexistence of alternative structures with different melting transitions. UV melting profiles performed for *E. coli* RPR revealed a major melting transition at 82°C (and minor ones at 57°C and 77°C), with the shape of the first derivative curve [65] being more similar to that of H1 RNA 6 than to those of H1 RNA variants 1, 3, and 5. This similarity is not unexpected, taking into account that *E. coli* RPR folds into a largely homogeneous stable secondary structure and H1 RNA 6 was engineered with multiple mutations mirroring the bacterial consensus, which increased the amount and stability of base‐pairing interactions in the C‐domain of the RPR.

## 3 Discussion

Our results show that changes toward the bacterial consensus in the C‐domain of H1 RNA could be coaxed into moderate activity improvements. In contrast to archaeal counterparts [26, 27, 29], we have not succeeded in activating the human C‐domain variants by fusing them to the S‐domain of *E. coli* or by covalent attachment of substrates, indicating that the catalytic defect of human H1 RNA is not simply a problem of low‐affinity substrate binding by its S‐domain. An explanation for the catalytic inactivity of HE chimeras could be that the *E. coli* S‐domain perturbed folding of the H1 RNA C‐domain, or the heterologous domains adopted inappropriate orientations towards each other preventing productive substrate binding. Likewise, covalent substrate attachment might have caused misfolding of the H1 RNA C‐domain.

H1 RNA 5 (mutations **A**), harboring a bacterial‐like P4 element, was the sole variant with locally confined mutations that showed moderately increased activity. This observation is consistent with the central role of P4 in various aspects of RNase P function (see Introduction). In the cryo‐EM structure of human nuclear RNase P, the N‐terminal domain of Pop1 binds to the single‐stranded region J7/19, between P4 and P7, which includes the conserved region CR‐IV that is part of the active site (Figure 1). Thereby, Pop1 stabilizes the conformation of the catalytic core structure through formation of extensive stacking and electrostatic interactions via several arginine residues [9]. Further stabilzations of the core structure are mediated by a long *α*‐helix in the C‐terminal domain of Pop1 that bridges P1 and P19, and by interactions of Pop1 and the Rpp20_Rpp25 heterodimer with the P3/P3′‐element [9]. Apparently, H1 RNA evolved a catalytic core structure that has become largely dependent on association with its natural protein cofactors for adopting an active conformation. Our mutations **A** toward the bacterial consensus in the P4 and J19/4 elements apparently slightly improved the capacity of the RNA to conduct metal‐ion dependent specific cleavage in the absence of its native protein cofactors. Notably, adaptations to a bacterial‐like P2_P3‐J3/4 structure (mutations **B** or **E**) or introduction of P5/P15 elements (mutations **C**) were somewhat disadvantageous, as was a combination of mutations **A** + **B**. Only combination of **A** + **B** + **C** improved H1 RNA activity more than mutations **A** alone. This finding underscores the idea that formation of a catalytically proficient RNA core structure is a highly cooperative process, in which the P4_J19/4 element appears to serve as a kind of seed structure for C‐domain folding including the formation of the P1/P4/P5 and P2/P3 helical stacks (for the crucial role of P4_J19/4, see also the Supporting Information). In addition, the P15 loop introduces an interaction site for the substrate CCA 3′‐end element, which may have improved substrate docking in the context of H1 RNA 6. In this context, an *E. coli* RPR variant that “mimics” the H1 RNA structure by deleting the P15‐P17 domain lowers the catalytic activity in the RNA‐alone reaction almost 50 000‐fold (based on *k*_obs_/*K* ^sto^ values) [21].

The bacterial P15 “domain”, encompassing elements P15–P17 in bacterial type A RPRs (see Figure S6) and P15‐P15.1 in type B RPRs, is of particular interest, as it was identified to be a minimal structural element capable of catalyzing RNase P‐type cleavage at rates similar to those of H1 RNA 1 [66]. The P15 “domain” can be considered as a cryptic active site with a different (and less efficient) mode of catalytic metal ion recruitment compared with full‐length RPRs. The domain shows activity in its isolated state, yet apparantly not in the context of full‐length RPRs, as an H1 RNA 1 variant with insertion of the *E. coli* elements P15–P17 was inactive [66].

We emphasize that even in the reaction catalyzed by the relatively most active H1 RNA 6, only a fraction (≈10%) of substrates was cleaved under single‐turnover conditions ([E] >> [S]) within the 22 h incubation period. From this perspective, a likely explanation for increased H1 RNA 6 activity is the presence of a moderately increased fraction of substrate binding‐competent and catalytically proficient H1 RNA 6 conformers.

In our study on reactivation of the archaeal C‐domain of type A RPR from *M. thermoautotrophicus* [26], we observed that mutations within a single structural element were in most cases insufficient to improve RNA‐alone activity. At least two alterations had to be introduced simultaneously to be beneficial for ribozyme activity. A key step for improving RNA‐alone activity of the archaeal C‐domain was adjustment of the P2/P3 region to the bacterial consensus (adjusting the length of P2 to 7‐bp and connecting P2 and P3 by a single G residue). This alteration (variant MM‐mJ2/3_P2; see Figure 2 in ref. [26]) caused a 135‐fold higher RNA‐alone activity relative to the parental archaeal P RNA, and acitvated a chimeric P RNA consisting of archaeal C‐ and *E. coli* S‐domain 450‐fold in the holoenzyme reaction with the *E. coli* RnpA protein [26]. However, applying the same strategy to H1 RNA was unsuccessful: introduction of a bacterial‐like P2/P3 region into H1 RNA 1 (Figure 1; variants H1 RNA 2 and 8) failed to improve catalytic performance. An obvious explanation for this difference is that the archaeal RPR, in contrast to H1 RNA, has retained a stable C‐domain structure, including a bacterial‐like P4_J19/4 element, despite recruitment of five different protein cofactors, rationalizing why the archaeal RPR has remained much more responsive to reactivation of RNA‐alone activity than H1 RNA. The robust structure of the archaeal C‐domain is suggested by the close similarity to bacterial type A RPR structures and was experimentally confirmed by the finding that substantial ribozyme activity could be regained (by at least two orders of magnitude) by a few minor structural changes towards the bacterial consensus [26]. The cryo‐EM structures of human nuclear and archaeal RNase P [8, 9] have confirmed that archaeal RNase P is intermediate between bacterial and eukaryal RNase P. Eukaryal‐like stabilization of RNA conformation by protein cofactors is already evident in archaeal holoenzymes, mainly for stabilizing the proper orientation of C‐ and S‐domain, but also for optimizing the conformation of the S‐ and C‐domain and formation of enzyme dimers. At the same time, bacterial‐like RNA‐mediated RNA stabilization is still prevalent in anchoring cleavage site and D/T‐loop of substrates to archaeal enzymes [8].

In the cryo‐EM structure of human nuclear RNase P, the *β*‐barrel of protein Rpp29 juxtaposes the terminal loop (L9) of helix P9 and the terminus of P1 [9]. In bacterial RPRs, this is achieved by the RNA loop‐helix interdomain contact between L9 and P1 [67]. Here we inserted *T. thermophilus* RPR elements P1/P9 (mutations **D**) into H1 RNA, intended to reinforce interdomain interactions as shown for the chimera of archaeal C‐ and *E. coli* S‐domain [29]. Yet, this impaired H1 RNA‐alone activity by two to nine fold, depending on the substrate, relative to H1 RNA 1. In this case, the domain interaction may have not been established owing to improper RNA folding. Alternatively, the contact formed but shifted the conformational equilibrium more toward inactive conformers, taking into account that Rpp29 may align the L9 and P1 elements in a somewhat different manner than the RNA‐mediated tetraloop‐helix contact. Another possibility is that the RNA L9‐P1 contact by itself is not stable enough for bridging C‐ and S‐domain of H1 RNA. This idea is consistent with our finding that the L18‐P8 tertiary contact, in addition to the L9‐P1 contact, had to form in the chimeric RPR consisting of an archaeal C‐ and bacterial S‐domain to obtain robust ribozyme activity in vitro and RNase P activity in *E. coli* cells [29].

Although associated with low RNA‐alone activity, the fold of H1 RNA can be considered as “globally optimized” since five of the structure variants, except for H1 RNA 5 and 6, had a lower activity than the parental H1 RNA 1 (Table 1) and impaired capability to productively interact with the *E. coli* RnpA protein (Figure 6). High sensitivity to structural alterations was previously also observed for another strongly protein‐dependent RNA, the A/U‐rich RPR of the *Cyanophora paradoxa* cyanelle [13]. Changes as little as a single point mutation or a base pair identity switch at positions that are not part of the universally conserved catalytic core led to a complete loss of RNA‐alone activity. Introduction of the entire P5‐7/P15‐17 subdomain from *E. coli* into the cyanelle RPR also abolished activity, but caused a structural compaction of this variant [13], similar to what we observed here in the comparative UV melting profiles when the bacterial P5/15 element was additionally introduced into H1 RNA (variant 6 vs. 3, Figure 8). We conclude that a group of eukaryotic RPRs, which have become increasingly protein‐dependent during evolution, can still adopt an active conformation to a very minor extent, but lost the capacity to autonomously stabilize their active RNA architecture such that a larger fraction of molecules populates the active state, which includes the recruitment and proper positioning of catalytic metal ions. As a consequence, attempts to regain substantial ribozyme activity for largely protein‐dependent RPRs by a limited set of specific local structural alterations are usually unsuccessful and rather diminish than increase the conformer fraction that adopts an active state.

The bacterial RnpA protein and the evolutionarily unrelated [68] eukaryal Pop5 protein were proposed to have overlapping binding modes in the RPR C‐domain where they stabilize the active C‐domain conformation and might interact with 5′‐leaders of precursor tRNAs in a similar manner [1, 9]. The RNA interface of both proteins includes multiple contacts to the universally conserved (see Figure S1) CR‐IV region (nt 272–276 in H1 RNA, PDB: 6ahr/6ahu; nt 332–337 in *E. coli* RPR, PDB: 7uo1; for nucleotide positions, see Figures 1 and S7). Compared with the RNA‐alone reaction, where H1 RNA 6 activity exceeded that of H1 RNA 5 (Figures 3 and 4 and Table 1), both H1 RNA variants showed roughly equal activities in the presence of RnpA at low Mg^2+^ concentration (10 mM). This equal activity gain for H1 RNAs 5 and 6 might be explained by a beneficial effect of mutations A (J19/4_P4) on RnpA binding, potentially by moderately enhancing the RNA's affinity for the protein or by increasing the fraction of RNA conformers that are able to productively interact with the bacterial RnpA protein. Furthermore, a G350C mutation in *E. coli* RPR (wild‐type H1 RNA has a C at this position) resulted in an activity defect in vitro and in vivo, assignable to a Mg^2+^ recruitment defect to the P4 region [69]. Possibly, this defect might be mitigated in H1 RNAs 5 and 6 with a G at the position corresponding to nt 350 in *E. coli* RPR. Substantially weaker, or no activation at all, in the case of H1 RNA 2–4, 7, and 8 (Figure 6) in the presence of the RnpA protein at 10 mM Mg^2+^ suggests that the mutations introduced into these RPR variants decreased the fraction of RNA conformers that are able to productively interact with the RnpA protein.

Our study can be seen as a promising initial effort to explore if and how a catalytic RNA that has almost entirely lost its ability to function without multiple protein cofactors can regain RNA‐alone acitvity. Thus, our investigation lays the foundation for future efforts to further enhance the ribozyme activity of H1 variants, possibly by combining our rational design approach with in vitro selection approaches.

## 4 Experimental Section

### 4.1 Construction of Plasmids for In Vitro Transcription

Plasmid‐based DNA templates for T7 in vitro transcription were constructed by standard molecular biology methods, detailed in the Supporting Information.

### 4.2 Primers for Plasmid Constructions

See Supporting Information, Table S1.

### 4.3 In Vitro Transcription and End‐Labeling of RNase P and Substrate RNAs

RNAs were prepared by T7 in vitro transcription as described [41, 70] using the following DNA templates: plasmid pSBpt3′HH linearized with BamHI for transcription of the *T. thermophilus* ptRNA^Gly^ substrate [71]; plasmid pJA2 linearized with FokI, yielding a 377‐nt transcript of *E. coli* RPR with native 5′‐ and 3′‐ends [58]; plasmid pUC19‐pATSerUG‐PstI linearized with PstI for synthesis of substrate pATSerUG [21]; all H1 RNA variants, HE chimeras and H1 RNA‐substrate conjugates were transcribed from pUC19 derivatives linearized with EcoRI, or, in rare cases, also directly from PCR fragments. 5′ and 3′ endlabeling with T4 polynucleotide kinase (*γ*‐[^32^]P‐ATP) and T4 RNA ligase (5′‐[^32^P]‐pCp), respectively, were performed essentially as described [41, 70]. T7 transcription was performed with guanosine as initiator nucleoside [41] to enrich for transcripts with 5′‐OH termini. After RNA purification by denaturing PAGE, RNAs were treated with calf intestine alkaline phosphatase (CIAP, MBI Fermentas) for the removal of 5′‐phosphates, as a fraction of transcripts was initiated with GTP. For this purpose, 200 pmol RNA were incubated with 10 U CIAP for 1 h at 37°C in 1 × CIAP buffer (50 mM Tris−HCl, 0.1 mM EDTA, pH 8.5 at 20°C) in a total volume of 0.5 ml, followed by phenol/chloroform (50%/50% v/v) extraction and ethanol precipitation. This procedure increased the 5′‐endlabeling efficiency (with *γ*‐[^32^]P‐ATP at 3000 Ci/mmol, 10 µCi [0.37 MBq]/µl) by a factor of 5 (to approximately 300 Ci/mmol RNA) relative to the procedure without CIAP treatment.

### 4.4 Pb^2+^‐Induced Hydrolysis

For Pb^2+^‐induced hydrolysis, we applied the experimental setup established for *E. coli* RPR that was used as the reference system [51]. RPRs to be investigated (250 nM including trace amounts [20 000 Cherenkov cpm] of 3′‐endlabeled RNA) were preincubated in 50 mM Tris‐HCl pH 7.5, 100 mM NH_4_Cl, 2 mM Mg(OAc)_2_ for 2 min at 70°C and then for 10 min at 37°C. The hydrolysis was initiated by adding a freshly dissolved Pb(OAc)_2_ solution to a final concentration of 0.5 mM. The reactions were incubated at 37°C for 6 min, terminated by mixing with loading buffer (10 M urea, 10 mM EDTA, 2% [w/v] bromophenol blue), and analyzed on an 8% (w/v) polyacrylamide (PAA) denaturing (7 M urea) sequencing gel. Two controls were prepared in parallel, one without Mg(OAc)_2_, the other without Mg(OAc)_2_ and Pb(OAc)_2_.

### 4.5 RNA‐Alone Processing Assays

5′‐[^32^P]‐labeled substrate (40 000–50 000 Cherenkov cpm per reaction and gel lane) was preincubated in buffer A (50 mM MES pH 6.0 [37°C], 800 mM NH_4_OAc, 160 mM Mg[OAc]_2_) for 5 min at 55°C and 25 min at 37°C; enzyme RNAs were preincubated in buffer A for 5 min at 55°C and 35 min at 37°C. For slow kinetics (single time points), 2 μl preincubated substrate mix (40 000–50 000 Cherenkov cpm) were combined with 8 μl preincubated RPR mix (final concentrations: 6 µM for H1 RNA variants, 370 nM *E. coli* RPR, approximately 5 nM substrate) followed by incubation for 22 h at 37°C (37°C for 30 min for *E. coli* RPR). RPR was omitted in control reactions incubated for the same time period. For the Mg^2+^ variation in Figure 4, buffer C (50 mM MES pH 6.0 at 37°C, 800 mM NH_4_OAc) was used and reactions were adjusted to the respective Mg^2+^ concentration. After the incubation step, samples were precipitated with ethanol and then analyzed by 22% (w/v) denaturing (7 M urea) PAGE (polyacrylamide gel electrophoresis), and quantified with a Bio‐Imaging Analyzer FLA 3000‐2R (Raytest, Fujifilm).

### 4.6 Processing Assays with *E. coli* RnpA Protein

For holoenzyme reactions, recombinant *E. coli* RnpA protein [72] was added to promote processing by H1 RNA variants at low salt conditions (specified below). Assays (final volume 12 µl) contained 200 nM of the H1 RNA variant or 10 nM *E. coli* (*Eco*) RPR and approximately 50 000 Cherenkov cpm (5 nM) of 5′‐^32^P‐endlabeled ptRNA^Gly^ or pATSerUG in buffer KN10 (20 mM Hepes pH 7.4 at 37°C, 2 mM spermidine, 0.05 mM spermine, 4 mM ß‐mercaptoethanol, 150 mM NH_4_OAc, and 10 mM Mg[OAc]_2_); the concentration of the *E. coli* RnpA protein was 1 µM when combined with H1 RNA variants or 50 nM with *E. coli* RPR. The entire incubation procedure was as follows: 8 µl of RPR mix in KN10 buffer was incubated for 15 min at 37°C, followed by addition of 2 µl RnpA protein solution and incubation for another 5 min at 37°C. Then 2 µl of substrate mix (approximately 50 000 Cerenkov cpm/µl of 5′‐^32^P‐endlabeled substrate), preincubated separately for 10 min at 37°C, was added to the 10 µl of enzyme mix to start the reaction. Reaction mixtures were incubated for 20 min (*Eco* RNA) or 4 h (H1 RNA variants) at 37°C. Control samples lacking any RPR or lacking both, RPR and RnpA protein, were incubated in the same manner. After incubation, reaction mixtures were subjected to ethanol precipitation, RNA pellets were dissolved in 4–8 µl of denaturing gel loading buffer (2.6 M urea, 66% (v/v) deionized formamide, 0.02% (w/v) each bromophenol blue and xylene cyanol blue, in 2 x TBE buffer, pH 7.5 to 8.0), and samples were analyzed by 22% (w/v) denaturing (7 M urea) PAGE.

### 4.7 Determination of Rate Constants (*k*_obs_ min^−1^)

Using the software PCBAS/AIDA for phosphorimage analysis, rate constants (*k*_obs_ min^−1^) were calculated as follows: rectangles of equal size were positioned on the unprocessed substrate bands, and another set of even rectangles on the 5′‐leader products derived from processing at the canonical RNase P cleavage site (assigned via comigration of a processing reaction catalyzed by *E. coli* RPR). The photostimulated luminescence (PSL) counts for the 5′‐leader were devided by the sum of PSL counts for substrate and 5′‐leader, yielding the fraction of cleaved substrate (*f*_cleaved_). The same was performed for the substrate‐alone (no H1 RNA added) control, yielding a “*f*_cleaved_ _(con)_” value that was subtracted from the *f*_cleaved_ value of the enzymatic reaction. This corrected *f*_cleaved_ _(corr.)_ was divided by 1320 min (22 h), yielding the *k*_obs_ (min^−1^) value.

### 4.8 UV Melting Profiles

UV melting profiles were recorded at 260 nm on a CARY 100 Bio UV–visible spectrophotometer, using the Cary Win UV software version 3.00. Experimental details and data analysis are described in the Supporting Information.

## Supporting Information

Additional supporting information can be found online in the Supporting Information section. The authors have cited additional references within the Supporting Information [73, 74, 75, 76]. **Supporting Fig. S1:** Phylogenetic minimum consensus secondary structures of bacterial and eukaryal RPRs, adapted from ref. [22]. **Supporting Fig. S2:** Mutant derivatives of H1 RNA Δ298C325 [21] illustrated in the context of the RPR secondary structure presentation of ref. [31]. For more information, see Figure 1 of the main text. The residue C325 (C323 in the numbering system used here) is marked in pink in the central structure; Δ298 is Δ297 in the numbering system of Fig. S2. **Supporting Fig. S3**: H1 RNA U325 in complex with human tRNA^Val^ in the cryo‐EM structure of human RNase P (PDB: 6ahu [9] with hidden protein cofactors, illustrated in three different orientations. The C298 deleted in H1 RNA Δ298C325 and the pyrimidine residue 325 (Py325) are highlighted in red. Structural elements that were mutated in the present study are indicated by different colors; structural elements that remained unchanged are shown in gray (P8, P19) or sand color (P10/11/12 domain). **Supporting Fig. S4**: Proposed secondary structures of circularly permuted H1 RNA‐substrate conjugates. Inserted nucleotides of the covalently linked substrate pATSerUG are highlighted by gray‐shading and red lettering; the inserted closing loop of helix P1 is highlighted by gray‐shading as well. **Supporting Fig. S5:** *Cis*‐cleavage assays of the H1 RNA‐substrate conjugates shown in Figure S3. Lanes *Eco*: processing of 5′‐^32^P‐labeled pATSerUG by *E.* *coli* RNase P RNA (RPR) as in Figure 3. The conjugates were analyzed in buffer C: 50 mM MES pH 6.0 (37°C), 800 mM NH_4_OAc) with or without 160 mM Mg(OAc)_2_. The reaction solution, containing ∼5 nM (40 000–50 000 Cherenkov cpm) of 5’‐endlabeld RNA, was heated at 55°C for 5 min, followed by incubation at 37°C for 22 h. Reactions were analyzed by 22% denaturing (7 M urea) PAGE. For H1‐pATSer‐5 and H1‐P15‐pATSer‐5, the size of the expected 5′‐cleavage product was expected to be the same as that obtained with *E.* *coli* RPR acting on pATSerUG (indicated by arrows). In the case of H1‐pATSer‐3, the 5′‐endlabeled conjugate was expected to be shortened by 36 nucleotides upon self‐cleavage. **Supporting Fig. S6:** Secondary structure of *E.* *coli* RPR. The border between catalytic (C) and specificity (S) domain is marked by the gray dashed line. Colored boxes connected by dotted lines indicate long‐range RNA tertiary interactions, with intradomain contacts in dark yellow and interdomain contacts in light blue and red brown (adapted from ref. [1]). Green arrows depict previously mapped Pb^2+^ hydrolysis sites [56, 63, 64, 76]. Prominent lead cleavage sites are indicated by Roman numerals, the most prominent ones being sites Ia and Ib. Hydrolysis site IVb (in red) is specific for RPR:tRNA complexes [56]. **Supporting Fig. S7:** Secondary structure of *E.* *coli* RPR in the presentation of ref. [31] (drawing on the left) and ref. [30] (drawing on the right). The catalytic (C) and specificity (S) domains are separated by the dashed lines. The conserved regions CR‐I, II, III, IV, and V are highlighted. Long‐range tertiary contacts are indicated by boxes connected by dotted lines. For futher details, see ref. [1]. **Supporting Table S1:** Primers for plasmid constructions. Underlined nucleotides indicate mutations to be introduced; Δ__ means a single nucleotide deletion. Restriction enzyme recognition sites for EcoRI and BamHI are highlighted in italics; T7 promoter sequences are depicted in lower case letters.

## Author Contributions

**Dan Li:** data curation (equal), formal analysis (equal), investigation (lead), methodology (lead), validation (equal), visualization (equal), writing – review & editing (supporting). **Leif A. Kirsebom:** conceptualization (supporting), funding acquisition (supporting), resources (supporting), supervision (supporting), validation (supporting), writing–review & editing (equal). **Roland K. Hartmann:** conceptualization (lead), data curation (equal), formal analysis (equal), funding acquisition (lead), project administration (lead), resources (lead), supervision (lead), validation (equal), visualization (equal), writing – original draft (lead), writing – review & editing (equal).

## Funding

This work was supported by Deutsche Forschungsgemeinschaft (GRK 1384, SPP 1170, and HA‐1672/7‐5); Vetenskapsrådet (VR 349‐2012‐1924, VR‐NT 521‐2009‐4535).

## Conflicts of Interest

The authors declare no conflicts of interest.

## Supporting information

Supplementary Material
