# Supplementary material for: Resurrecting the Activity of the RNA Subunit of Human RNase P
Source: Chembiochem. 2026 Mar 26;27(6):e202500803. doi: 10.1002/cbic.202500803 (PMC13022451; doi:10.1002/cbic.202500803)
Supplement: Supplementary file 1 — Supplementary Material [file CBIC-27-e202500803-s001.pdf]

Supporting Information

Resurrecting human RNase P RNA (H1 RNA) alone activity

Dan Li[1, 2], Leif. A. Kirsebom[3] and Roland K. Hartmann[1,*]

1 Institut für Pharmazeutische Chemie, Philipps-Universität Marburg, D-35037 Marburg, Germany

2 Current address: College of Pharmaceutical Sciences, Zhejiang University, Yuhangtang Road 866, 310058 Hangzhou, Zhejiang, People's Republic of China,
E-mail: lidancpu@zju.edu.cn

3 Department of Cell and Molecular Biology, Biomedical Center, Uppsala University, Box 596, SE-751 24 Uppsala, Sweden, E-mail: Leif.Kirsebom@icm.uu.se

[*]Corresponding author: Roland K. Hartmann, Institut für Pharmazeutische Chemie, Philipps-Universität Marburg, Marbacher Weg 6, D-35037 Marburg, Germany,
Tel. +49-6421-2825827; Fax +49-6421-2825854; e-mail: roland.hartmann@staff.uni-marburg.de

EXPERIMENTAL PROCEDURES

Construction of plasmids for *in vitro* transcription

The plasmid pUC19-Hsa-Δ298C325 for H1 RNA transcription was constructed in several steps. The *H. sapiens* H1 RNA gene was first amplified from HeLa genomic DNA as a 430-bp fragment with primers 224 and 225 and cloned into the pCR®2.1-TOPO® vector with the TOPO TA® cloning kit (Thermo Fisher Scientific [TFS] Invitrogen, formerly Life Technologies Invitrogen). The H1 RNA structural gene was then amplified with primer pair 233 and 234, digested with BamHI and EcoRI and ligated into pUC19 cut with the same restriction enzymes. Then the two site mutations, Δ298 and C325, were introduced by two-step site-directed *Dpn*I mutagenesis according to the protocol of the QuikChange XL Site-Directed Mutagenesis Kit (Agilent Technologies Stratagene). Primers 241 and 242 introduced the C325 mutation, and primers 243 and 244 the G298 deletion into plasmid pUC19-Hsa, yielding pUC19-Hsa-Δ298C325. To generate plasmid pUC19-Hsa-Δ298C325-mut-P5/P15, a two-step cloning strategy was applied. In the first step, one part of the mutation P5/P15 was inserted into pUC19-Hsa-Δ298C325 by "inside-out"-PCR mutagenesis[13] with primers 245 and 246. In the second step using the template of the first mutagenesis product, another "inside-out"-PCR mutagenesis inserted the other part of the mutation P5/P15 by use of primers 247 and 248. The plasmids pUC19-Hsa-Δ298C325-mut-P2-3_J3/4 and pUC19-Hsa-Δ298C325-mut-P4_J19/4 were both produced by "inside-out"-PCR mutagenesis with primer pairs 251/252 and 257/256, respectively, using pUC19-Hsa-Δ298C325 as template. The plasmid pUC19-Hsa-Δ298C325-mut-P2-4_J19/4 combined the mutations P4_J19/4 and P2-3_J3/4. "Inside-out"-PCR mutagenesis with primers 257 and 256 added the mutation P4_J19/4 to plasmid pUC19-Hsa-Δ298C325-mut-P2-3_J3/4. For the construction of plasmid pUC19-Hsa-Δ298C325-mut-P1_P9, the DNA sequence parts encoding *Thermus thermophilus* P9 were first introduced into pUC19-Hsa-Δ298C325 by "inside-out"-PCR mutagenesis with primers 258 and 254. One unwanted mutation in pUC19-Hsa-Δ298C325-mut-P9 was corrected by site-directed *Dpn*I mutagenesis with primers 279 and 280. Based on the corrected pUC19-Hsa- Δ298C325-mut-P9 as template, the *T. thermophilus* P1 element was introduced by PCR with primers 271 and 272, followed by digestion with BamHI and EcoRI and cloning into pUC19, resulting in construct Hsa-Δ298C325-mut-P1_P9. Because primer 271 introduced another EcoRI site immediately downstream of the T7 promoter, EcoRI could not be used for plasmid linearization in T7 transcription reactions; instead, primer pair 271/272 was used to amplify a PCR fragment from the sequence-verified plasmid Hsa-Δ298C325-mut-P1_P9, which served as template for T7 transcription. For construction of plasmid pUC19-Hsa-Δ298C325-mut-P3, the *Geobacillus stearothermophilus* P3 element was introduced in pUC19-Hsa-Δ298C325 by "inside-out"-PCR mutagenesis with primers 273 and 274, which simultaneously introduced three point mutations as present in mutant P2-3_J3/4. The permuted enzyme-substrate conjugate Hsa-Δ298C325-pATSerUG-5 was constructed in several PCR steps. First, three PCRs were done separately to amplify the segments of the pATSerUG substrate coding region (primers: 261 and 262;

template: pUC19-pATSerUG-PstI), the 5’ part of the H1 RNA gene (primers 263 and 264; template: pUC19-Hsa-Δ298C325) as well as the 3’ part of H1 RNA gene (primers 265 and 266; template: pUC19-Hsa-Δ298C325). PCR products 1 and 2, and 2 and 3 overlapped with each other. The three PCR fragments were purified by agarose gel electrophoresis and then combined for overlap extension PCR. In this PCR, primers 261 and 266 amplified the final product, which was digested with EcoRI and BamHI, and cloned into pUC19 to yield plasmid pUC19-Hsa-Δ298C325-pATSerUG-5. Accidental mutations in the substrate portion were corrected with primers 283 and 284 by “inside-out”-PCR mutagenesis. The same strategy was applied to construct pUC19-Hsa-Δ298C325-pATSerUG-3. The 5’ part of the H1 RNA gene (primers: 267 and 264; template: pUC19-Hsa-Δ298C325), the 3’ part of the H1 RNA gene (primers: 265 and 268; template: pUC19-Hsa-Δ298C325) as well as the pATSerUG coding region (primers: 269 and 270; template: pUC19-pATSerUG-PstI) were amplified separately. Using the three gel-purified PCR fragments as templates, overlap extension PCR with primers 267 and 270 amplified the full-length construct that was subsequently cloned into pUC19. Finally, a coincidental mutation was corrected by “inside-out”-PCR mutagenesis using primers 281 and 282. Similarly, as was performed for pUC19-Hsa-Δ298C325-mut-P5/P15, two “inside-out”-PCR mutagenesis reactions were carried out to obtain pUC19-Hsa-Δ298C325-P15-pATSerUG-5 using plasmid pUC19-Hsa-Δ298C325-pATSerUG-5 as template, with primers 291 and 292 in the first reaction, and primers 289 and 290 in the second reaction. Primers 293 and 294 were used to correct one captured mutation by “inside-out”-PCR mutagenesis. A modification of the megaprimer mutagenesis technique[73] was employed to substitute the *E. coli* S-domain for the *Homo sapiens* S-domain in plasmid pUC19-Hsa-Δ298C325 (primers 295 and 296), pUC19-Hsa-Δ298C325-mut-P4_J19/4 (primers 295 and 296), pUC19-Hsa-Δ298C325-mut-P2-4_J19/4 (primers 301 and 296), and pUC19-Hsa-Δ298C325-mut-P2-5_P15_J19/4 (primers 302 and 303). The megaprimers were amplified in each case from pHY300-EE [13] using the primers just mentioned. All constructed sequence variants were validated by Sanger sequencing (Eurofins).

UV melting analyses

A Cary 100 Bio UV-Visible spectrophotometer (VARIAN) was used, including a heating block with drillings for 12 cuvettes. Before measurements, the heating block was prewarmed for at least 30 min. The software "Thermol" (Cary Win UV software version 3.0) was initiated, and the measuring program and parameters were set in the module "Setup". "Wavelength" was set to 260 nm, "SBW" (required spectral bandwidth) to 1.0 nm and "Ave Time" to 2.000 s. The measuring program is specified below.

Measuring program

Stage	Data Interval (°C)	Rate (°C/min)	End (°C)	Hold (min)
1	1.00	10.00	55.00	5.00
2	1.00	10.00	37.00	35.00
3	0.10	0.50	90.00	0.00
4	0.10	2.00	25.00	5.00

Stages 1 to 3 comprised a single round of measurement, with stages 1 and 2 representing preincubation steps to support RNA folding; stage 1: heating of the sample to 55°C at a rate of 10°C/min, incubation for 5 min at 55°C, cooling of the sample to 37°C at a rate of 10°C/min, incubation at 37°C for 35 min. In stage 3, the UV melting curve was generated by heating the sample from 37°C to 90°C at a rate of 0.5°C/min, with data collecting every 0.1°C. For additional measurement rounds, the sample was immediately cooled to 25°C (stage 4) after 90°C had been reached, and then subjected to stages 1 to 3 again. For providing the data in Excel format, "Select for ASCII (CSV)" in the subdirectory of Setup/Reports/autoconvert was chosen.
For melting curve measurement of H1 RNAs, similar buffer conditions as for H1 RNA-alone kinetic assays were adopted: 2 mM EDTA, 50 mM MES pH 6.0 and 100 mM NH_4OAc (corresponding to 1 x melt buffer), plus [Mg^{2+}] confined to 4.5 mM because high concentrations of divalent metal ions resulted in aberrant signals at high temperature.

Sample for melting curve measurement, 150 µl

	Sample	C_{end}	Control
5 x melt buffer	30 µl	1 x	30 µl
45 mM $Mg(OAc)_2$	15 µl	4.5 mM	15 µl
RNase-free water	90 µl		105 µl
2.5 µM P RNA	15 µl	250 nM	---

Samples (150 µl each) were pipetted into a standard cuvette (micro cell, 80 µl, 4 mm x 10 mm stoppered, VARIAN). The surface of the sample mix was covered using 200 µl of PCR-grade mineral oil to prevent evaporation and condensation at high temperature. Then each cuvette was firmly sealed with a stopper. The cuvettes were placed into heating slots of the multicell holder, with a blank containing water or measuring buffer placed in slot 1. The lid of the machine was closed. The button “Zero” within the “Thermol” menu was clicked for the blank. The measurement was initiated by clicking “Start”.

Cleaning of the cuvette

To clean the cuvettes after measurement, the sample solution and mineral oil were removed with a pipette. The cuvette was rinsed once with 70 % ethanol followed by three times with double-distilled water. Then 2% cuvette wash solution (HELLMANEX®II, HELLMA) was filled into the cuvette, followed by incubation for a few minutes. Subsequently, the cuvette was washed another three times with double-distilled water. Finally, the remaining liquid was removed with a syringe and the cuvette was left to dry upside down on a piece of clean tissue paper.

Data analysis

Data analysis was accomplished with the software “Thermol”. In the mode “Maths”, after selecting a trace or a graph, one could choose an operation such as “Mean”, “Deriv” (the first derivative, d Abs/d T), “Normalize” or “Smooth” for the curves; operations had to be confirmed by clicking “Apply” and then “=”. Usually, the mean curve was calculated from at least three measurements for each RNA species, normalized to 89 (°C) for the X-axis and to 1.0 (normalized Abs) for the Y-axis, followed by conversion to the first derivative and final curve smoothing. To combine several curves that had not been measured simultaneously, the curves were saved as Data [^{x}.DTM] files, then opened together by ticking “Overlay Data” and using the key “Strg” on the computer keyboard, followed by saving as a Batch [^{x}.BTM] file. Choosing curves and changing their colors was done in the mode “Trace preferences”. After normalization, each RNA species displayed a specific UV melting profile which could be reliably reproduced in different rounds of experiments. The major melting transition was inferred manually by clicking the peak on the first derivative curve.

Table S1: Primers for plasmid constructions

Underlined nucleotides indicate mutations to be introduced; Δ means a single nucleotide deletion. Restriction enzyme recognition sites for EcoRI and BamHI are highlighted in italics; T7 promoter sequences are depicted in lower case letters.

No.	sequence
224	5′-CTGTATGAGACCACTCTTTCCC-3′
225	5′-CGGTTTCGTTTTGTTTTTTTTTTTTTTTTTTTTTCCAAA-3′
233	5′-GCG*GGATCC*taatacgactcactataggATAGGGCGGAGGGAAGCTC-3′
234	5′-CGC*GAATTC*AATGGGCGGAGGAGAGTAGTCT-3′
241	5′-GACCTCATAACCCAATTCAGACCACTCTCCTCCGCCCATTTTTG-3′
242	5′-CAAAAATGGGCGGAGGAGAGTGGTCTGAATTGGGTTATGAGGTC-3′
243	5′-GCTGAGGTGAGGTACCCCGΔAGGGGACCTCATAACCCAATTC-3′
244	5′-GAATTGGGTTATGAGGTCCCCTΔCGGGGTACCTCACCTCAGC-3′
245	5′-ATGGTAGGGGCTCAGATCAATGGCTGAGGTGAGGTAC-3′
246	5′-CATTTGGGTTΔGCTCCGGCCGTGAGTCTGTTCCAAGC-3′
247	5′-CACTAGGGCCAGAGGCGGCCCTAAC-3′
248	5′-GAGCTCAGACCTTCCCAAGGGACATG-3′
251	5′-CTGTCACTCCACTCCCATGTCCCTTGAGGAAGGTCTGAGACTAGGGCCA-3′
252	5′-GACGCACTCAGCTCGTGGCCCCTGCGGCTGAGCTTCCCTCCGCCCT-3′
254	5′-TACCCCGCAGCCCTGTTAGGGCCGC-3′
256	5′-AGTTCTGTTATGAGGTCCCCTCGGGGTA-3′
257	5′-CAGΔCCTACTCTCCTCCGCCCATTGAATTCA-3′
258	5′-ACCCGACGTGAGTTCCCAGAGAACG-3′
261	5′-CGC*GGATCC*taatacgactcactatagGATCTGAATGGAGAGAGGGGGT-3′
262	5′-GCCATTGAACTCACTACCGGATCCTGGCGGA-3′
263	5′-GATCCGGTAGTGAGTTCAATGGCTGAGGTGAGGT-3′
264	5′-GATCGAGTAAATCAGGTGAAAATGGGCGGAGGAGAGTGGGT-3′
265	5′-TTCACCTGATTTACTCGATCATAGGGCGGAGGGAAGCTCATCAG-3′
266	5′-CGC*GAATTC*TTCGCTGGCCGTGAGTCTGTT-3′
267	5′-CGC*GGATCC*taatacgactcactataggAATGGCTGAGGTGAGGTACCCCG-3′
268	5′-GATCGCCATAACTCACTTCGCTGGCCGTG-3′
269	5′-GAAGTGAGTTATGGCGATCTGAATGGAGAG-3′
270	5′-CGC*GAATTC*TGGCGGAGAGAGGGGGAT-3′
271	5′-CGC*GGATCC*taatacgactcactatagGGGAATTCCGGGACGAGGGCTCATCAG TGGGGCCAC-3′
272	5′-CGC*GAATTC*GCCTCCGGGACGAGGCGTGGTCTGAATTGGGTTATGA-3′

273	5′-CGGTTTCGGCCGTAGAGGAAGGTCTGAGACTAGGGCCAGA-3′
274	5′-GCCGCAGCGGCTGAGCTTCCCTCCGCCCTAT-3′
279	5′-GCCCTAACAGGGCTGCGGGGTAACCCGACGTGAGTTCCCAGAG-3′
280	5′-CTCTGGGAACTCACGTCGGGTTACCCCGCAGCCCTGTTAGGGC-3′
281	5′-ΔGAGGGGGTTCAAATCCCCCTCT-3′
282	5′-TCTCCATTCAGATCGCCATAACTCA-3′
283	5′-TCTCCGCCAGCATGCGGTAGTGAGTTCAATGGCTGAGGTG-3′
284	5′-GAGGGGGATTTGAACCCCCTCTCTCCATTCAGATCCTATAG-3′
289	5′ATCGGCGTGAGTTCAATGGCTGAGGT-3′
290	5′-ACGGAGAGAGGGGGATTTGA-3′
291	5′-AGAATTCACTGGCCGTCGTTTTAC
292	5′-ATTTCGGCTTCGCTGGCCGTGAGTCTGTTC-3′
293	5′-GTTCAAATCCCCCTCTCTCCGTATCGGCGTGAGTTCAATGGCTGAGG-3′
294	5′-CCTCAGCCATTGAACTCACGCCGATACGGAGAGAGGGGGATTTGAAC-3′
295	5′-CTCCCATGTCCCTTGGGAAGGTCTGAGACTAGGGGTGCCAGGTAACGCCTG-3′
296	5′-GTACCTCACCTCAGCCATTGAACTCACTTCGCTGGGTGGAGTTTACCGTG<br>CCA-3′
301	5′-CTCCCATGTCCCTTGAGGAAGGTCTGAGACTAGGGGTGCCAGGTAACGC<br>CTG-3′
302	5′-CTCCCATGTCCCTTGAGGAAGGTCTGAGCTCCACTAGGGGTGCCAGGTAACGC<br>CTG-3′
303	5′-GCCATTGATCTGAGCCCCTACCATCATTTGGGTTΔGCTCCGGGTGGAGTTT<br>ACCGTGCCA-3′

Background information on the structural alterations introduced into H1 RNA Δ298C325 (= H1 RNA 1)

Mutations **A** (mut P4_J19/4; H1 RNA 5)

The bacterial-like helix P4 was designed to form 8 Watson-Crick base pairs (bp) with a central U bulge; in addition, two nucleotides in J19/4 were adapted to the bacterial consensus. X-ray structures of bacterial RPRs[32, 33] suggested that the universally conserved P4 helix and its adjoining single-stranded regions in the core are crucial for stabilizing the functional conformation of the RNA. This expectation is consistent with previous biochemical studies indicating a key role of P4 in many aspects of RNase P function, such as positioning of divalent metal ions[34-36], substrate binding and catalysis[37-42].

Mutations **B** (mut P2-3_J3/4. H1 RNA 2)

Mutations **B** (mut P2-3_J3/4) restored a bacterial-like P2-P3 subdomain, which included shortening of the J2/3 junction to a single G residue. P2 and the bottom part of P3 were extended to 7 and 6 bp, respectively. An A residue was inserted in J3/4 in line with the bacterial consensus. The P2/P3 region, in addition to being important for the function of the catalytic RNA, is located at the bacterial RnpA protein binding surface[24, 26, 43, 44]. Mutations **B** were introduced into H1 RNA to analyze if this alteration improves RNA-alone function and possibly interaction with the bacterial RnpA protein.

Mutations **C** (mut P5/P15, H1 RNA 4)

Mutations **C** introduced P5 and P15 with bacterial-like flanking sequences as found in type B (for *Bacillus*-like) RPRs, representing a structurally simpler version than those present in type A RPRs. Helix P5 and junction J5/15 were identical to the corresponding sequence of *E. coli* RPR. Helix P15 and loop L15 are based on the *G. stearothermophilus* RPR (type B). The lack of P5 and P15 is one of the most obvious distinctions between eukaryal RPRs and bacterial counterparts. In bacterial RPRs, the two modules positioned in the core structure are important for overall conformation, binding of substrate via the 3'-CCA end, Mg^{2+} binding as well as RnpA interaction[24, 45-48]. The absence of P5 and P15 is also assumed to contribute to the high flexibilty of the H1 RNA core structure.

Mutations **D** (mut *T. th.* P1/P9, H1 RNA 7)

L9-P1 is one of the loop-helix interdomain contacts in bacterial RPRs. In the archaeal type A RPR from *Methanothermobacter thermoautotrophicus*, the presence of the natural (extended) P1 helix resulted in a 200-fold increase in the RNA-alone activity compared with an RNA variant carrying a shortened P1 helix[26], providing evidence for the formation of the L9-P1 contact in this archaeal RPR. Also, swapping the P1 and P9 modules of a thermostable RPR (*T. thermophilus*) for those of *E. coli* converted the chimeric *E. coli* RPR to a thermostable ribozyme[67], demonstrating the capacity of this interaction to stabilize an active tertiary fold. Likewise, the same structural alteration was implemented in a chimera consisting of an archaeal C-domain and *E. coli* S-domain to make this chimera function in *E. coli* [29]. We therefore simultaneously introduced the P1 and P9 modules of *T. thermophilus* RPR into H1 RNA (mutations **D**) to reinforce this interdomain contact (H1 RNA 7, mut *T. th.* P1/P9).

Mutations **E** (mut *G. st.* P3, variant H1 RNA 8)

One of the prominent structural features of eukaryal RPR is the P3 element harbouring a large internal loop. The P3 element of bacterial RPRs is positioned beside P15 in the crystal structures[24]. Transfer RNAs carrying photoreactive groups at the 3’-end crosslinked to the internal (type A RPRs) or apical (type B RPRs) loop of P15 of bacterial RPRs, and to the P3 internal loop of the eukaryotic *Schizosaccharomyces pombe* RPR[25, 74]. Thus, in 3D models of eukaryal RPR, the internal loop of P3 is located close to the region where P15 is positioned in bacterial RPRs[25], as later verified by the cryo-EM structure[9]. Since the eukaryal P3 domain, particularly its enlarged central bulge, requires proteins Pop6 (yeast nomenclature; Pop6 corresponds to Rpp25 in human RNase P) and Pop7 (Rpp20 in human RNase P) for structural organization[9, 75], we replaced it with P3 of *G. stearothermophilus* RPR (mutations **E**) to restore an entirely bacterial-type P2-3_J3/4 region.

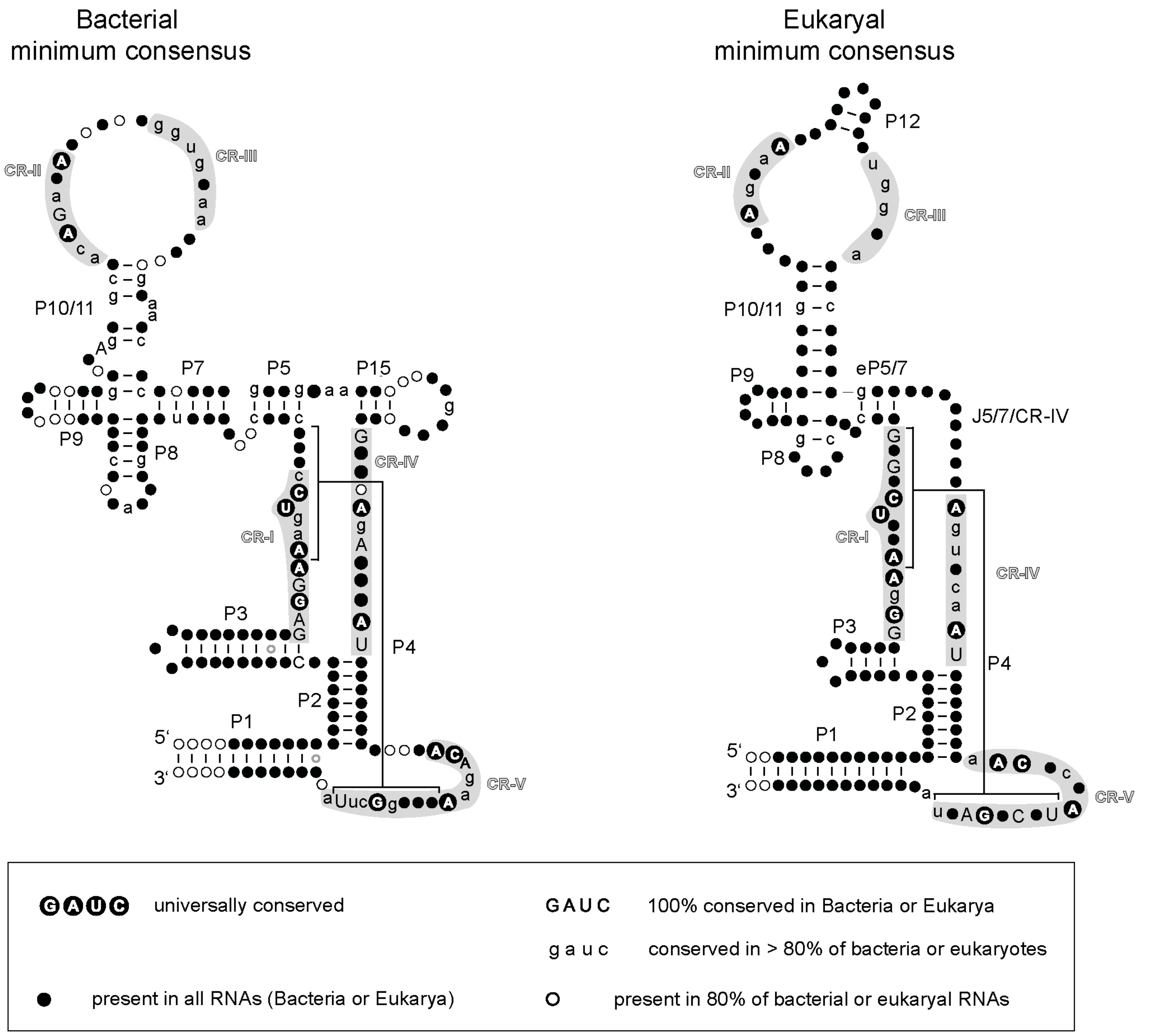

Fig. S1: Phylogenetic minimum consensus secondary structures of bacterial and eukaryal RPRs, adapted from ref. [22].

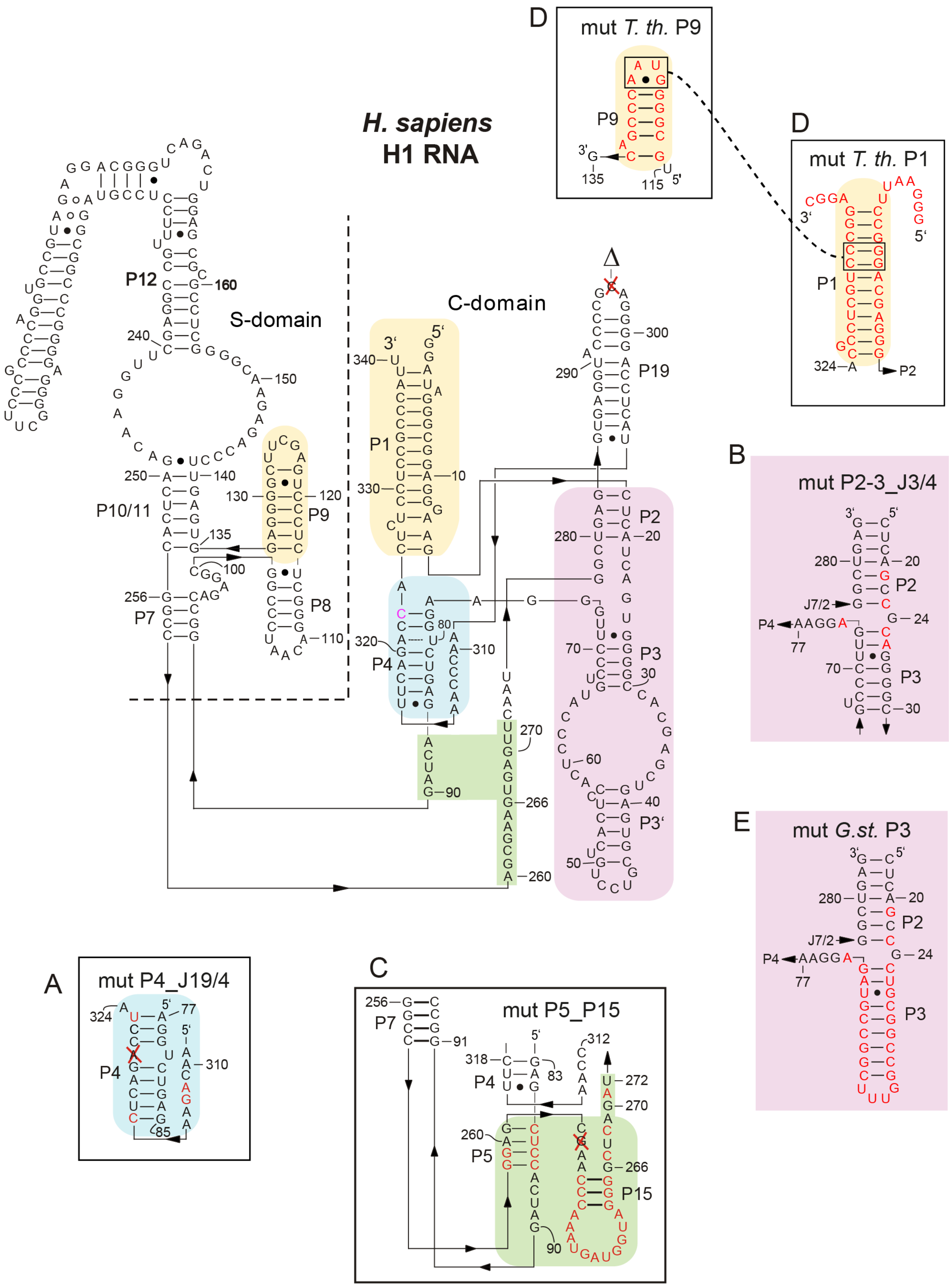

Fig. S2: Mutant derivatives of H1 RNA Δ298C325 [21] illustrated in the context of the RPR secondary structure presentation of ref. [31]. For more information, see Fig. 1 of the main text. The residue C325 (C323 in the numbering system used here) is marked in pink in the central structure; Δ298 is Δ297 in the numbering system above.

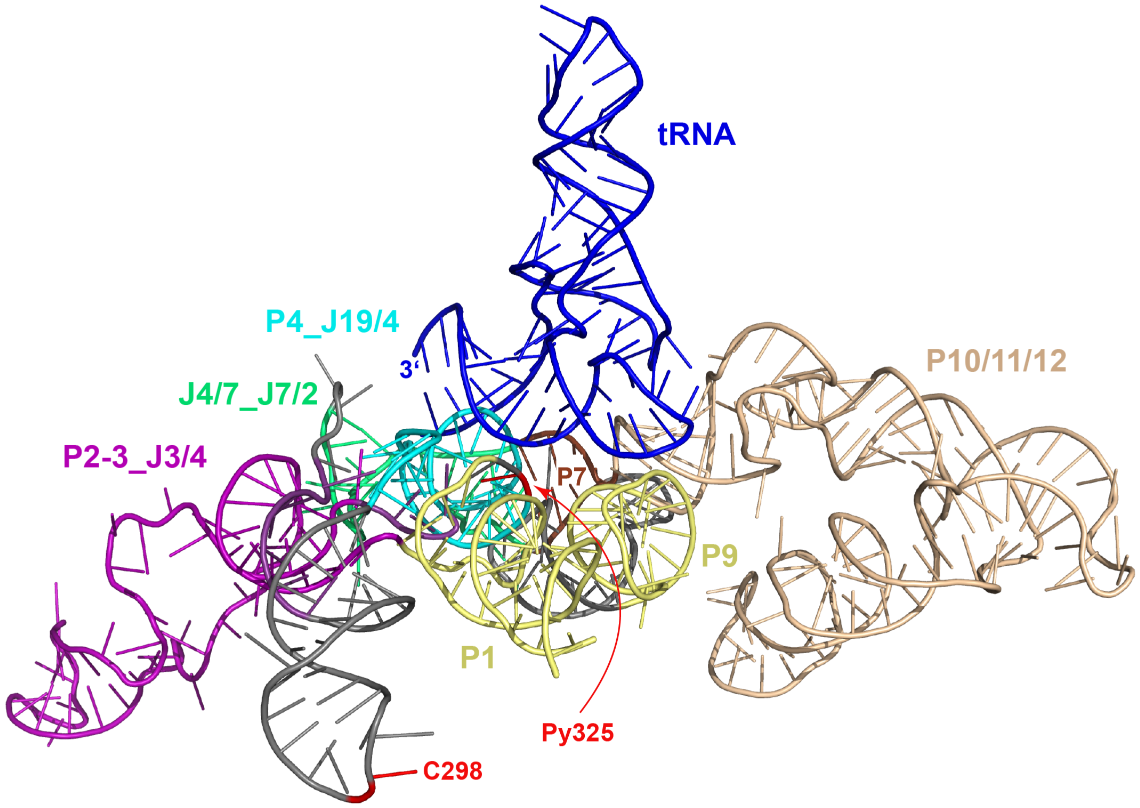
tRNA
P4_J19/4
3‘
J4/7_J7/2
P10/11/12
P2-3_J3/4
P7
P9
P1
Py325
C298

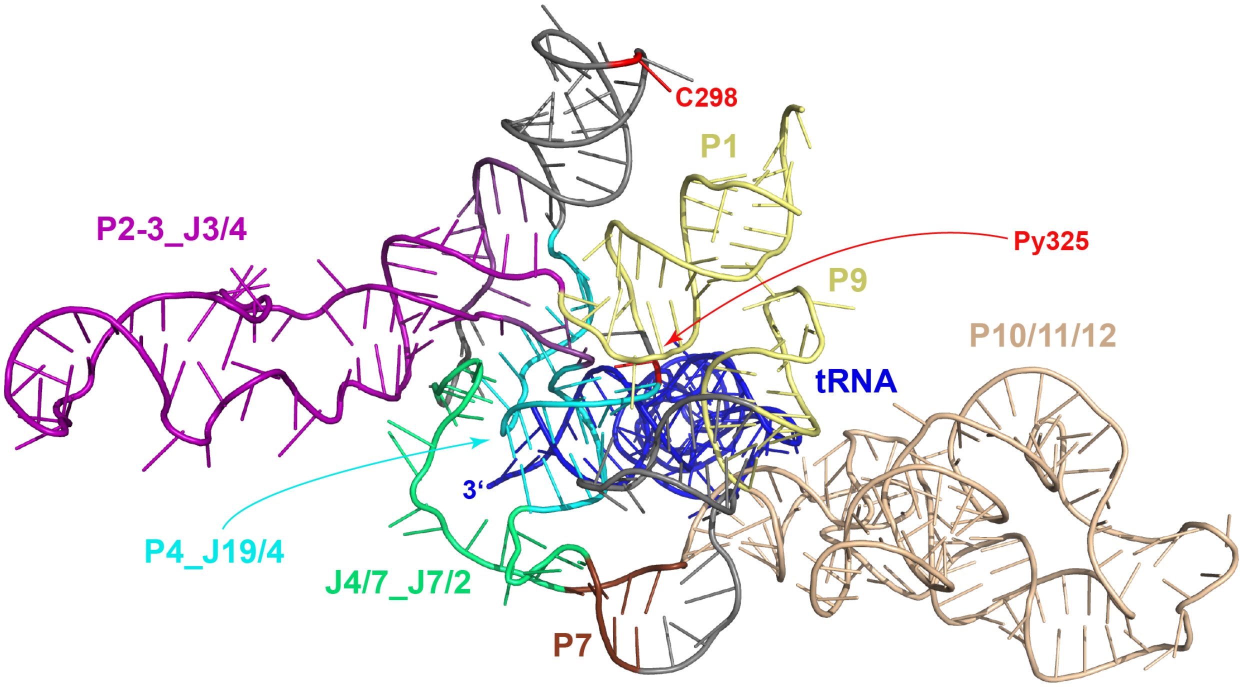
C298
P1
P2-3_J3/4
Py325
P9
P10/11/12
tRNA
3‘
P4_J19/4
J4/7_J7/2
P7

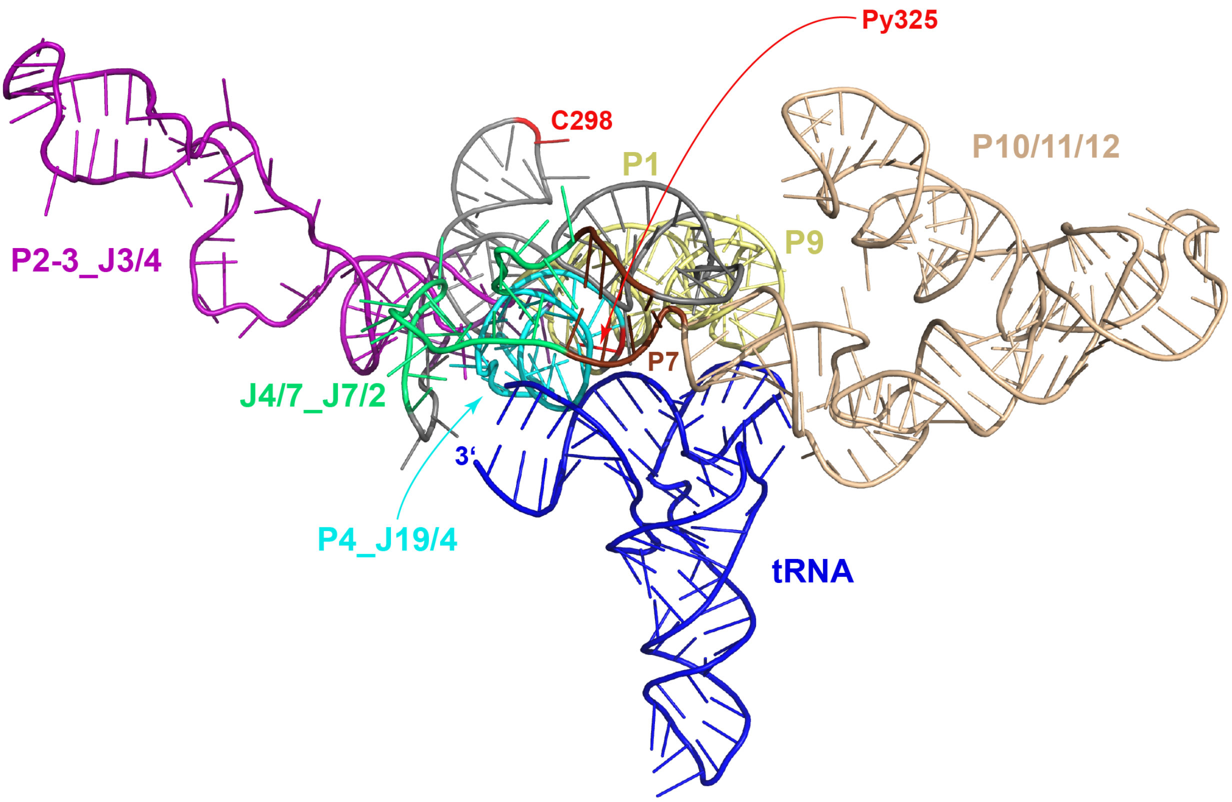

Fig. S3: H1 RNA U325 in complex with human tRNA^{Val} in the cryo-EM structure of human RNase P (PDB: 6ahu [9] with hidden protein cofactors, illustrated in three different orientations. The C298 deleted in H1 RNA Δ298C325 and the pyrimidine residue 325 (Py325) are highlighted in red. Structural elements that were mutated in the present study are indicated by different colors; structural elements that remained unchanged are shown in gray (P8, P19) or sand color (P10/11/12 domain).

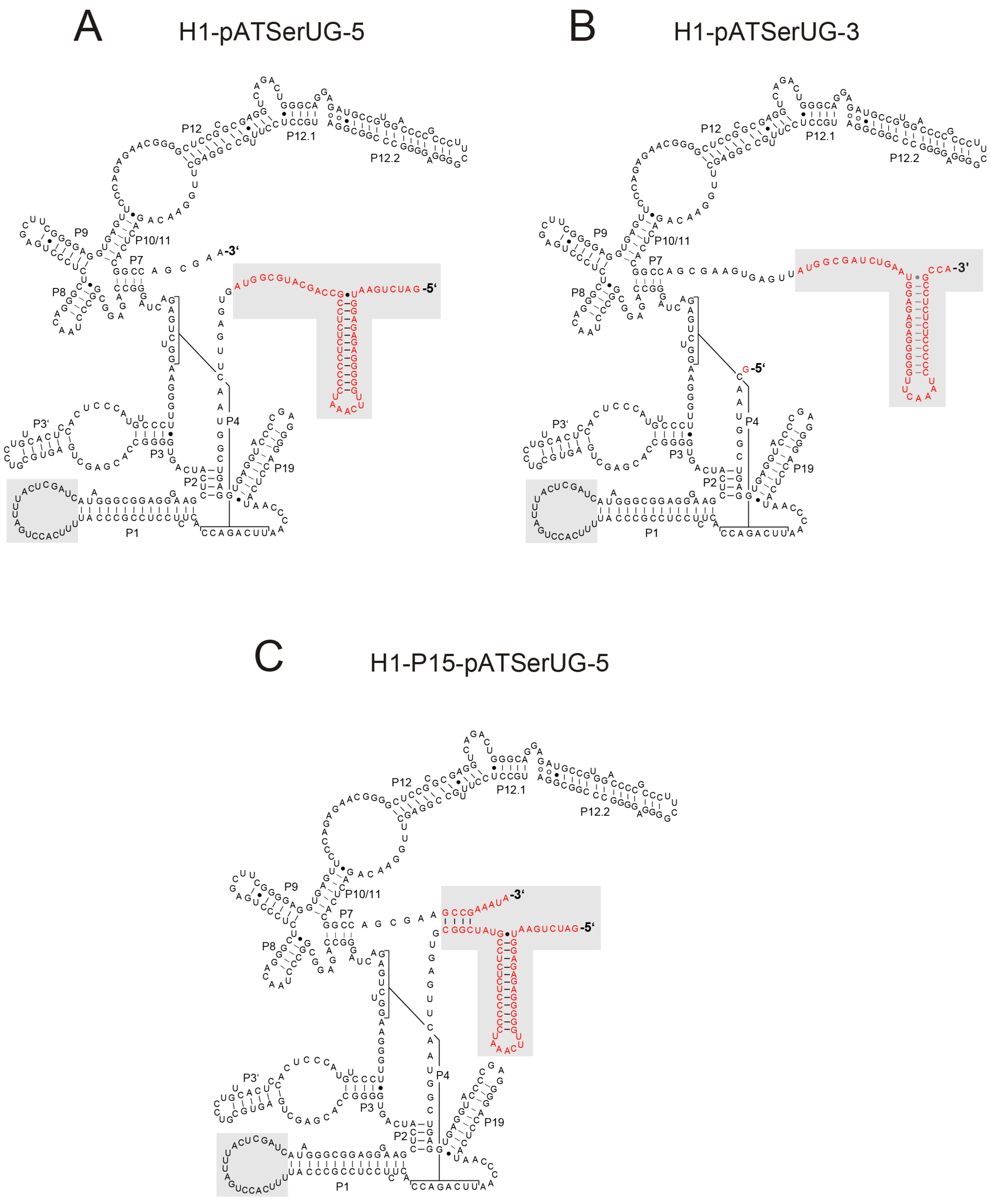

Fig. S4: Proposed secondary structures of circularly permuted H1 RNA-substrate conjugates. Inserted nucleotides of the covalently linked substrate pATSerUG are highlighted by gray-shading and red lettering; the inserted closing loop of helix P1 is highlighted by gray-shading as well.

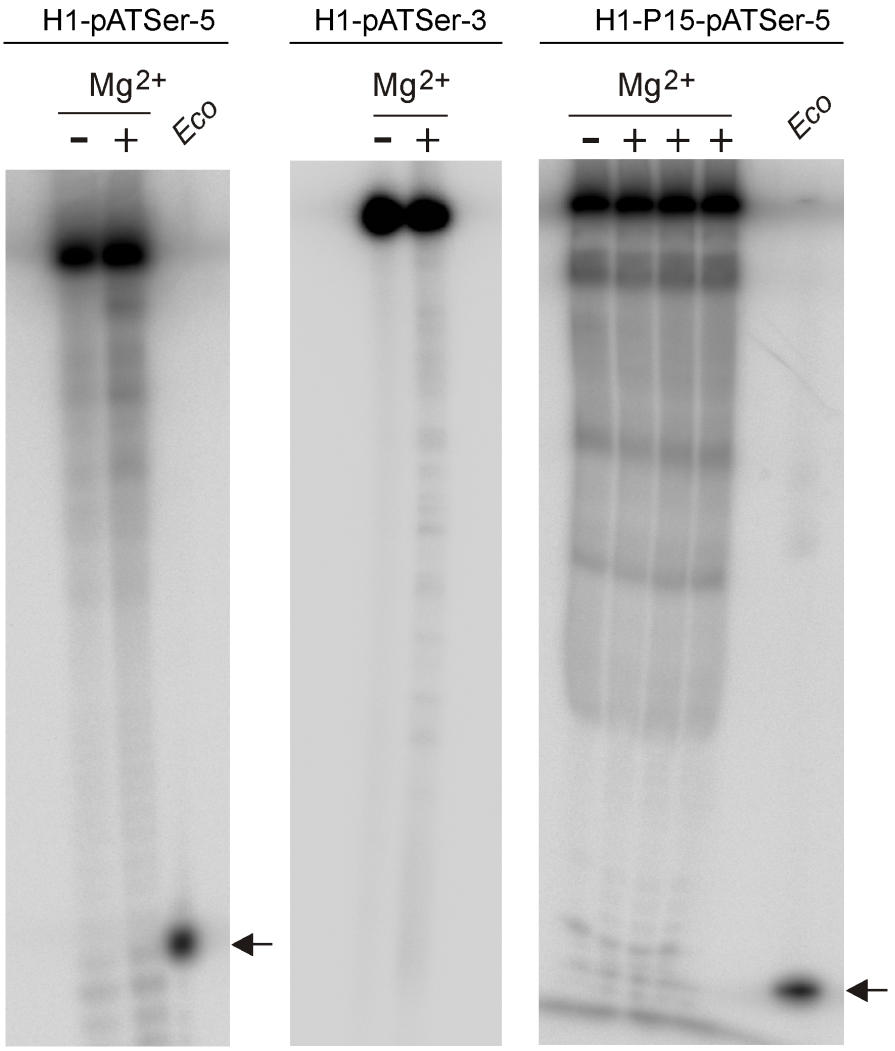

Fig. S5: *Cis*-cleavage assays of the H1 RNA-substrate conjugates shown in Fig. S3. Lanes *Eco*: processing of 5’-^{32}P-labeled pATSerUG by *E. coli* RNase P RNA (RPR) as in Fig. 3. The conjugates were analyzed in buffer C: 50 mM MES pH 6.0 (37°C), 800 mM NH_4OAc) with or without 160 mM $Mg(OAc)_2$. The reaction solution, containing ~5 nM (40,000-50,000 Cherenkov cpm) of 5’-end labeld RNA, was heated at 55°C for 5 min, followed by incubation at 37°C for 22 h. Reactions were analyzed by 22% denaturing (7 M urea) PAGE. For H1-pATSer-5 and H1-P15-pATSer-5, the size of the expected 5’-cleavage product was expected to be the same as that obtained with *E. coli* RPR acting on pATSerUG (indicated by arrows). In the case of H1-pATSer-3, the 5’-end labeled conjugate was expected to be shortened by 36 nucleotides upon self-cleavage.

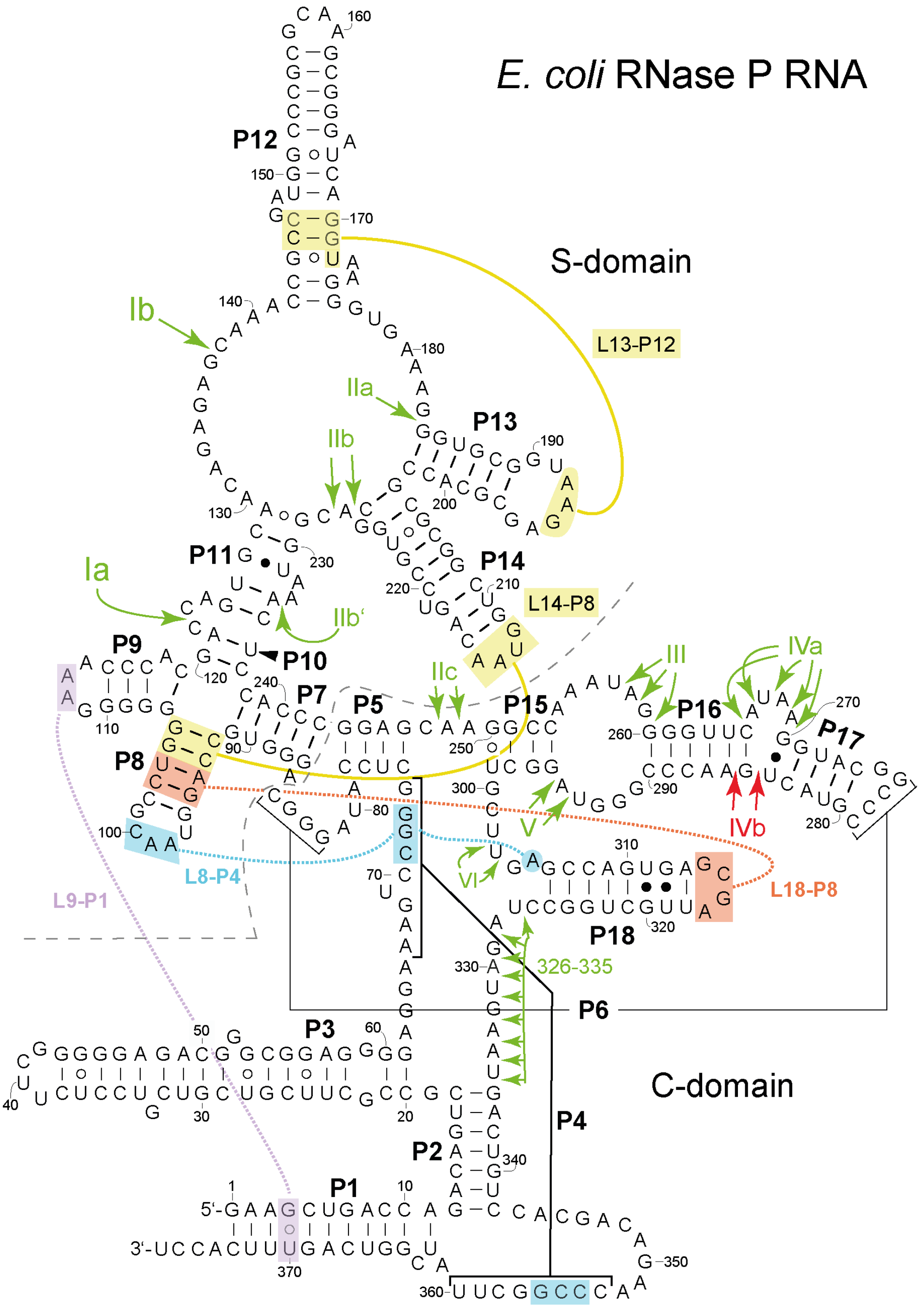

Fig. S6: Secondary structure of *E. coli* RPR. The border between catalytic (C) and specificity (S) domain is marked by the gray dashed line. Colored boxes connected by dotted lines indicate long-range RNA tertiary interactions, with intradomain contacts in dark yellow and interdomain contacts in light blue and red brown (adapted from ref. [1]). Green arrows depict previously mapped Pb^{2+} hydrolysis sites[56, 63, 64, 76]. Prominent lead cleavage sites are indicated by Roman numerals, the most prominent ones being sites Ia and Ib. Hydrolysis site IVb (in red) is specific for RPR:tRNA complexes[56].

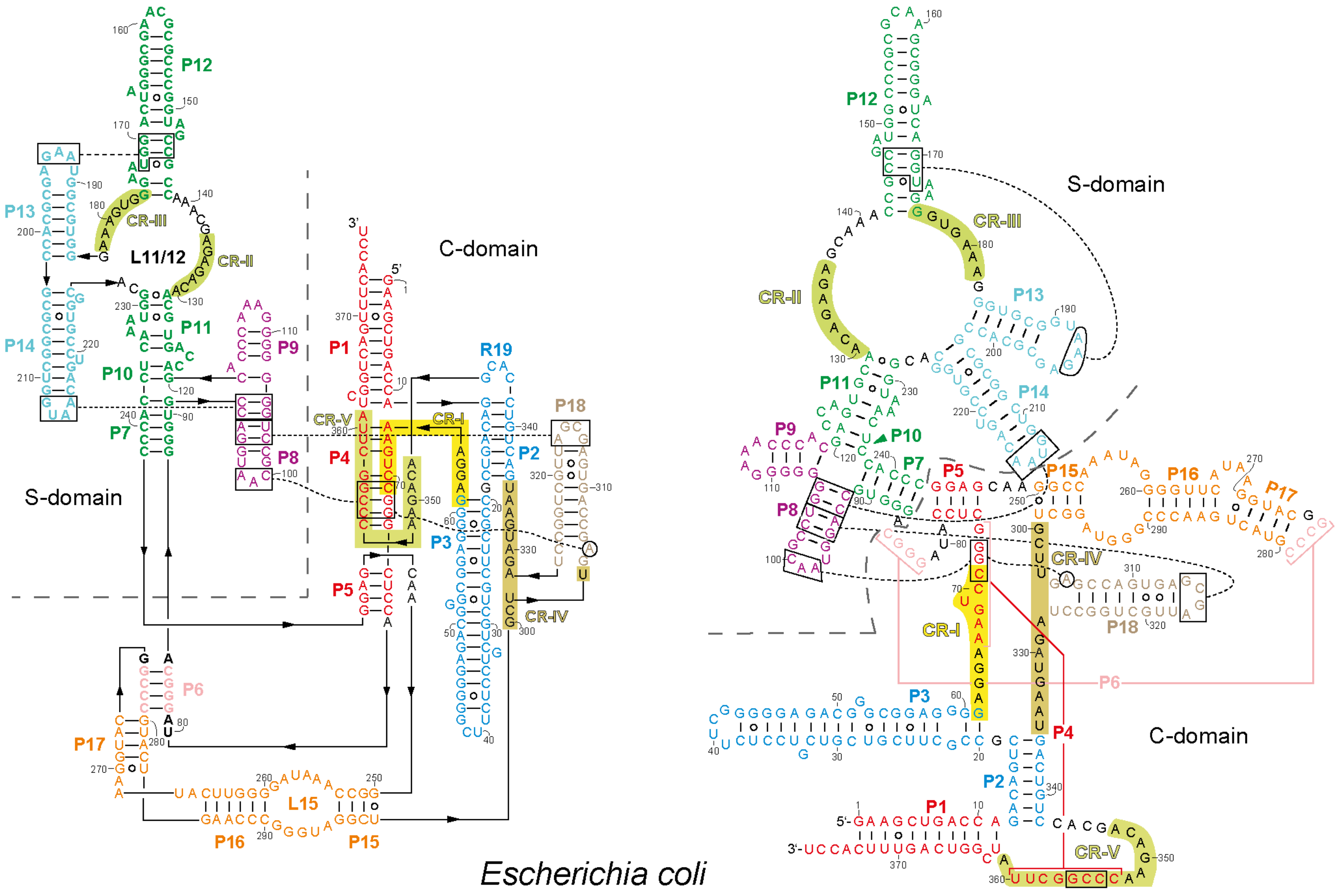

Fig. S7: Secondary structure of *E. coli* RPR in the presentation of ref. [31] (drawing on the left) and ref. [30] (drawing on the right). The catalytic (C) and specificity (S) domains are separated by the dashed lines. The conserved regions CR-I, II, III, IV, and V are highlighted. Long-range tertiary contacts are indicated by boxes connected by dotted lines. For futher details, see ref. [1].
